# Identifying key underlying regulatory networks and predicting targets of orphan C/D box *SNORD116* snoRNAs in Prader–Willi syndrome

**DOI:** 10.1093/nar/gkae1129

**Published:** 2024-11-22

**Authors:** Rachel B Gilmore, Yaling Liu, Christopher E Stoddard, Michael S Chung, Gordon G Carmichael, Justin Cotney

**Affiliations:** Department of Genetics and Genome Sciences, University of Connecticut School of Medicine, Farmington, CT, 06030, USA; Institute for Human Genetics, Heidelberg University Hospital, Heidelberg, BW, 69120, Germany; Department of Genetics and Genome Sciences, University of Connecticut School of Medicine, Farmington, CT, 06030, USA; Department of Genetics and Genome Sciences, University of Connecticut School of Medicine, Farmington, CT, 06030, USA; Department of Genetics and Genome Sciences, University of Connecticut School of Medicine, Farmington, CT, 06030, USA; Department of Genetics and Genome Sciences, University of Connecticut School of Medicine, Farmington, CT, 06030, USA; Department of Genetics and Genome Sciences, University of Connecticut School of Medicine, Farmington, CT, 06030, USA; Department of Surgery, Children's Hospital of Philadelphia, Philadelphia, PA, 19104, USA

## Abstract

Prader-Willi syndrome (PWS) is a rare neurodevelopmental disorder characterized by neonatal hypotonia, followed by hyperphagia and obesity. Most PWS cases exhibit megabase-scale deletions of paternally imprinted 15q11-q13 locus. However, several PWS patients have been identified harboring much smaller deletions encompassing the *SNORD116* gene cluster, suggesting these genes are direct drivers of PWS phenotypes. This cluster contains 30 copies of individual *SNORD116* C/D box small nucleolar RNAs (snoRNAs). Many C/D box snoRNAs have been shown to guide chemical modifications of RNA molecules, often ribosomal RNA (rRNA). Conversely, *SNORD116* snoRNAs show no significant complementarity to rRNA and their targets are unknown. Since many reported PWS cases lack their expression, it is crucial to identify the targets and functions of *SNORD116*. To address this we modeled PWS in two distinct human embryonic stem cell (hESC) lines with two different sized deletions, differentiated each into neurons, and compared differential gene expression. This analysis identified a novel set of 42 consistently dysregulated genes. These genes were significantly enriched for predicted *SNORD116* targeting and we demonstrated impacts on FGF13 protein levels. Our results demonstrate the need for isogenic background comparisons and indicate a novel gene regulatory network controlled by *SNORD116* is likely perturbed in PWS patients.

## Graphical Abstract

**Graphical Abstract:**
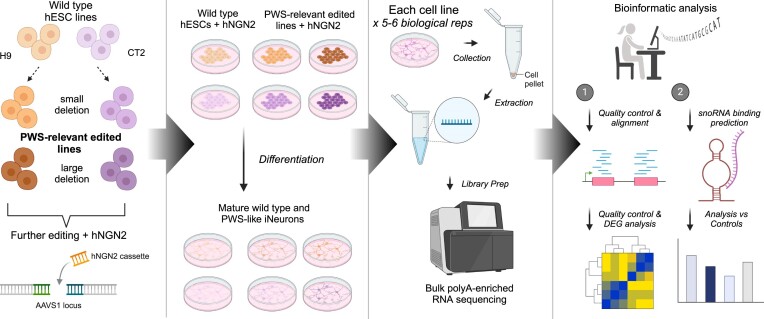

## Introduction

Prader–Willi syndrome (PWS [OMIM #176 270]) is a rare, neurodevelopmental disorder characterized by neonatal hypotonia and failure to thrive during infancy, followed by hyperphagia and obesity; small stature, hands, and feet; mild to moderate cognitive deficit; and a range of behavioral and sleep problems (1–3). PWS is linked to instability of chromosome 15 at locus 15q11-q13 that can result in inheritance of a variety of chromosomal structural changes (4,5). The most common structural change in PWS patients is the loss of several megabases of the 15q11-13 locus specifically on the paternally inherited allele. This is due to the fact that many genes in this region are imprinted, a phenomenon in which genes are expressed exclusively from one parental allele. This imprint is established in the germline via DNA methylation on the maternal allele at the Prader–Willi Syndrome Imprinting Center (PWS-IC) (6–8). The PWS-IC is also a promoter for a complex transcriptional unit that includes protein-coding genes (SNURF and SNRPN), many species of small nucleolar RNAs (*SNORD65*, *SNORD108*, two copies of *SNORD109*, 30 copies of *SNORD116* and 48 copies of *SNORD115*), antisense RNA that can silence *UBE3A* (*UBE3A-ATS*), and other RNA species that are not well understood (9–15). In addition to DNA methylation at the PWS-IC, post-translational methylation modifications have also been found in this chromosomal region. Zinc finger protein ZNF274 has been found to bind specifically to SNORD116 DNA sequences (16,17)). This binding event is thought to recruit lysine methyltransferases SETDB1 and EHMT2 (18,19)) which results in deposition of methylation marks on lysine 9 of histone H3 (H3K9me3), an epigenetic mark frequently associated with heterochromatin and gene silencing. There are other protein coding genes that are also imprinted as this locus, *MKRN3*, *MAGEL2* and *NDN*, but are positioned upstream of the PWS-IC and governed by different promoter sites. Notably, mutations in *MAGEL2* cause Schaaf-Yang syndrome (SYS [OMIM #615 547]), another rare neurodevelopmental disorder which shares some phenotypes with PWS (20) (https://www.ncbi.nlm.nih.gov/books/NBK567492/).

While megabase-scale deletions are the most common genetic subtype of PWS, a handful of patients have been reported to have atypical microdeletions (21). These deletions specifically affect the tandem array of 30 copies of *SNORD116. SNORD116* is a member of the C/D box class of small nucleolar RNAs (snoRNAs). *SNORD116* can be further subdivided into three subgroups based on sequence similarity: Group I (*SNORD116-1* to *SNORD116-9*), Group II (*SNORD116-10* to *SNORD116-24*), and Group III (*SNORD116-25* to *SNORD116-30*) (12,22)). While sometimes referred to as *SNOG1*, *SNOG2* and *SNOG3*, *SNORD116* groups will be referred to henceforth as *SNORD116*-I, *SNORD116*-II and *SNORD116*-III for clarity. snoRNAs are generally thought to be processed by exonucleolytic trimming from the introns of a host gene (23) and serve as a scaffold and specificity factor for ribonucleoprotein complexes that deposit 2′-O methylation on maturing ribosomal RNAs (rRNAs) (24). However, *SNORD116*, as well as the other snoRNAs found in the 15q11-13 region, do not have sequence complementarity to rRNA. Thus, it is unclear if they participate in rRNA maturation and are typically referred to as orphans (25). A previous study utilized the BLASTn algorithm to predict *SNORD116* sites transcriptome wide (26). However, only a handful of predicted targets were interrogated in HeLa cells making it unclear if they are relevant for PWS.

Since the function of *SNORD116* thus far has remained elusive, much effort has recently been expended to identify gene expression patterns that are dysregulated in PWS. Several studies have compared gene expression between tissue or cell lines derived from PWS patients and those from unrelated controls (27–31). While each of these studies identified numerous genes with distinct expression patterns in the PWS context, a coherent set of consistently dysregulated disease relevant genes has not been identified. Inherent differences in genetic background or postmortem delay may obscure important gene expression changes, leading to lack of a consensus set of perturbed genes in the disorder. Therefore, we have turned to the use of isogenic human embryonic stem cell (hESC) lines, to provide a more rigorous approach to investigate cellular deficits in disease models. Here, we describe the generation of two distinct hESC lines, each engineered with two separate deletions relevant to determining the targets and functions of *SNORD116* snoRNAs. We also utilized an inducible Neurogenin-2 (NGN2) expression system to enable quick, reproducible differentiation of these lines into neurons (32). Performing bulk RNA-sequencing on resulting neurons allowed us to identify a novel list of 42 genes consistently transcriptionally dysregulated in our PWS-like systems. Importantly, our results showed it is critical to use multiple isogenic cell line pairs as this eliminated many spuriously differentially expressed genes (DEGs). Employing the recently described computational tool snoGloBe (33), we discovered these dysregulated genes are significantly enriched for predicted *SNORD116* targeting versus multiple control analyses. Our results indicate a novel gene regulatory network controlled by *SNORD116* is likely perturbed in PWS patients.

## Material and methods

### Genome editing of hESCs

H9 ESCs were first engineered with a deletion of the entire *SNHG14* transcript (lgDEL) or *SNORD116* alone (smDEL) and then subsequently edited to introduce a neurogenin-2 (NGN2) cassette into the AAVS1 locus following the protocol described below.

#### Preparation

Guide RNAs for the lgDEL and smDEL were designed using available guide RNA design tools (Supplemental Table S1). Each guide was cloned into the pSpCas9(BB)-2A-Puro (PX459) V2.0 plasmid, a gift from Feng Zhang (Addgene, #62 988). This plasmid was digested with Bbs1 restriction enzyme and ligated with the guide RNA insert. Two days prior to planned genome editing, a 100 mm dish of mitotically inactivated DR4 mouse embryonic fibroblasts (MEFs) was prepared. hESCs were gown on mitotically inactivated MEFs and fed daily with sterile-filtered DMEM/F12 media (Gibco, # 11 330 032) supplemented with 20% Knock Out Serum Replacement (Gibco, #10 828 028), 1X MEM Non-essential amino acids (Gibco, #11 140 050), 1 mM L-glutamine (Gibco, #25 030 081) with 0.05 mM β-mercaptoethanol and 8 ng/mL bFGF (Gibco, #PHG0023), until ∼60–75% confluent. Cells were treated with 10 μM ROCK inhibitor, Y-27632 2HCl (Tocris, #1254), 24 h prior to planned genome editing.

#### Nucleofection

The day of editing, approximately 1–1.5 × 10^6^ cells were treated with Accutase (Millipore, #SCR005) to release the cells from the plate, cell suspension was singularized by pipetting, and then pelleted. The media was removed from the cell pellet and cells were resuspended according to the protocol provided for the P3 Primary Cell 4D-Nucleofector Kit (Lonza, #V4XP-3024). Briefly, a mixture of 82 μL nucleofector solution, 18 μL nucleofection supplement, and desired plasmids were added to the pellet. The pellet was resuspended in the solution by pipetting gently three times using a P200 pipet. For the smDEL and lgDEL edits, 2.5 μg of each CRISPR plasmid was added to the nucleofection solution (Supplemental Table S1). For the introduction of the NGN2 cassette, 2 μg of both TALEN-L and TALEN-R plasmids (Addgene, #59 025 and #59 026) and 4 μg of pUCM-AAVS1-TO-hNGN2 plasmid (Addgene, #105 840) was added to the nucleofection solution. The cell suspension was transferred to the nucleofection cuvette and nucleofection was performed using the 4D-Nucleofector (Lonza) on the program for hESC, P3 primary cell protocol. After nucleofection, hESC suspension was transferred to the 100 mm dish plated with DR4 MEFs containing the KOSR media mentioned above and supplemented additionally with 10 μM ROCK inhibitor using the transfer pipet included in the Lonza kit. For the NGN2 edit, the media of the 100 mm dish was also supplemented with 5 μM L755507 (Selleckchem, #S7974) and 1 μM SCR7 (Selleckchem, #S7742) to encourage homology directed repair for incorporation of the NGN2 cassette into the AAVS1 locus.

#### Selection

For lgDEL and smDEL edits, feeding media was changed 24 h following transfection (Day 1 post-transfection) and supplemented with 1 ng/μL puromycin and 10 μM ROCK inhibitor. This selection was continued for 48 h total to select cells transiently expressing the vectors containing the gRNA and Cas9 protein. On Day 2, the media was changed and supplemented with fresh 1 ng/μL puromycin and ROCK inhibitor. On Day 3, the media was changed and supplemented with fresh ROCK inhibitor. Subsequent media changes occurred every other day, supplemented with fresh ROCK inhibitor. Once small colonies became visible, media changes occurred daily with fresh media alone. After a total of 15 days, each colony was manually passaged into its own well of a 24-well plate coated with mitotically inactivated MEFs via cutting and pasting. Feeding media in the 24-well plate was supplemented with 10 μM ROCK inhibitor to encourage cell attachment. After 48 h of passaging cells, the feeding media was changed. Approximately 4 days after passaging to a 24-well plate, a few colonies from each well were isolated into polymerase chain reaction (PCR) tube strips and pelleted for screening.

For the NGN2 edit, feeding media was changed 24 h following transfection supplemented with fresh 10 μM ROCK inhibitor, 5 μM L755507 and 1 μM SCR7. Between 72–96 h post-transfection, selection began by supplementing fresh feeding media with 1 ng/μL puromycin and 10 μM ROCK inhibitor. Selection continued for 4 or 5 days by changing feeding media and supplementing with fresh 1 ng/μL puromycin. After selection, colonies were grown to a size sufficient for clonal isolation. Each colony was manually passaged into its own well of a 24-well plate coated with mitotically inactivated MEFs via cutting and pasting. After approximately one week of growth, a few colonies from each clone were manually passaged to a new 24-well plate. The remaining colonies from each clone were transferred to a 1.5-mL microcentrifuge tube and pelleted for screening.

#### Screening

For lgDEL and smDEL edits, DNA was extracted using the HotSHOT method (34). In brief, media was removed from pelleted cells and 30 μL of alkaline lysis buffer (25 mM NaOH, 0.2 mM EDTA, pH ∼12) was added to each tube. The tubes were incubated at 95°C for 45 min. Subsequently, 30 μL of neutralization reagent (40 mM Tris-HCl, pH ∼5) was added to each tube. Tubes were capped tightly, flicked to mix, and spun down.

For the NGN2 edit, genomic DNA (gDNA) was extracted using a homemade lysis buffer containing 1% SDS, 75 mM NaCl, 25 mM EDTA and 200 μg/mL Proteinase K. Briefly, 500 μL of the lysis buffer was added to each cell pellet and the tubes were incubated at 63°C overnight. The following day, 170 μL of 150 mM NaCl was added, followed by the addition of 670 μL of chloroform. The mixture was shaken vigorously (∼60 times) and centrifuged at 11 000 rcf for 10 min at room temperature. The top aqueous layer (∼650 μL) was removed and transferred to a new tube to which an equal amount of 100% isopropanol was added. The mixture was shaken ∼10 times and was incubated at −20°C for 20 min. Next, the mixture was centrifuged at 20 000 rcf for 20 min at 4°C. The supernatant was removed, and the pellet was washed with 70% ethanol before being resuspended in 50 μL of 10 mM sterile-filtered Tris.

Genotyping was performed using the Herculase II Fusion DNA Polymerases kit (Agilent, #600 677) following manufacturer's protocol. For the DNA template, 1 μL of each sample was used per 25 μL reaction. The annealing temperature was 60°C for all primer combinations (Supplemental Table S1). The PCR products were run on a 1% agarose gel for 35 min at 95V. Primer sets were designed upstream of the 5′ CRISPR cut site and downstream of the 3′ CRISPR cut site for each lgDEL and smDEL edits. For lgDEL and smDEL clone screening, first PCR primers for knockout of the region of interest were utilized (lgDEL or smDEL, primer set 1,2). If there was successful knockout on one or both alleles, a band would be present. Any clones identified as positive for a knockout then were screened using PCR primers to identify heterozygous clones (lgDEL and smDEL, primer set 1,2). For a heterozygous clone, a band would be present. RNA was extracted from heterozygous clones and subjected to cDNA synthesis using the SuperScript First-Strand Synthesis System for RT-PCR (Invitrogen, #11 904 018) following manufacturer's protocol to test for the parent-of-origin of the deleted allele. Finally, RT-PCR was performed on the cDNA with primers for *SNORD116* and a control, *GAPDH* (Supplemental Table S1). Clones which did not express *SNORD116* were then further expanded and banked down. One such clone from each genotype was subsequently edited for incorporation of NGN2. For NGN2 edits, a nested PCR across the insertion sites was used to identify clones which NGN2 was incorporated into the AAVS1 locus in the correct orientation. These primers were designed so that one primer was in the endogenous AAVS1 locus and the other primer was in the exogenous transgene which allows only clones with the insertion of the transgene into the correct locus in the correct orientation to be detected. Following the first PCR (PCR1, using primer sets 1,2 and 3,4), a second ‘nested’ PCR (PCR2, using primer sets 1,2 and 3,4) was run utilizing the product from PCR1 as the template for PCR2. Final nested products from both primer sets with banding at ∼1 kb indicated successful incorporation of the NGN2 cassette. Clones with correct NGN2 cassette integration were further screened for heterozygosity utilizing primers for the wild type AAVS1 locus (primer set 1,2). Wild type or heterozygous clones showed a band at 500 bp. Clones that showed homozygous insertion of NGN2 were expanded and banked down.

#### Confirmatory testing

An off-target analysis of CRISPR guides was performed. Briefly, off targets were determined using Cas-OFFinder (http://www.rgenome.net/cas-offinder/) (35) with default settings except for up to three mismatches allowed for each gRNA sequence excluding the PAM site. Primers were then designed using primer3plus to flank the off-target sites (Supplemental Table S1). PCR was performed on ∼150 ng of template gDNA from each sample using the Herculase II Fusion Enzyme with dNTPs Combo Kit (Agilent, #600 677) for each primer combination (Supplemental Table S1). An annealing temperature of 60°C for all primer sets and a total reaction volume of 25 μL was used. PCR cleanup was performed on PCR products using QIAquick PCR Purification Kit (QIAGEN, #28 106) following manufacturer's instructions for centrifuge processing. Purified PCR products were eluted in UltraPure water and sent for Sanger sequencing (Quintara Biosciences) using the forward PCR primer (Supplemental Table S1). Sequences were aligned using Clustal Omega on SnapGene software (v.5.3.3). One clone from each genotype and background, verified for correct genomic editing was used for the sequencing experiment described. Additionally, gDNA from edited clones and wild type controls was analyzed by CytoSNP array (Illumina Infinium®, CytoSNP-850K with BeadChip v1.2) through the University of Connecticut Chromosome Core to check for large copy number variations.

### hESC culture

To transition cells from feeder conditions to feeder-free conditions, cells were manually passaged by cutting and pasting colonies once confluent. After 5–7 days, any differentiation was manually removed before first passage. Routine culture of H9 and CT2 ESCs was done using feeder-free conditions. Cells were maintained in mTeSR™ Plus media (STEMCELL Technologies, #100–0276) on Matrigel™ hESC-Qualified Matrix (Corning™, #354 277) coated 6-well plates in a humidified atmosphere with 5% CO_2_ at 37°C. Feeding media was changed daily. Cells were passaged once 80–100% confluency was reached, approximately every 4–5 days. Briefly, media was removed from well(s), well(s) were gently rinsed with sterile PBS, sterile filtered 0.5 mM EDTA in PBS was added to well(s), and the plate was placed back into the incubator undisturbed for 2–5 min. After incubation, EDTA solution was gently aspirated from well(s), being careful to not disturb cells. Using a 2-mL serological pipette, 1 mL of media was added to well(s) while gently scraping bottom of well(s) to dislodge cells. The cell suspension was pipetted 1–2 times to break up the cells into clumps. Around 75–125 μL of cell suspension was added to a new well containing 2 mL of culture media supplemented with 10 μM ROCK inhibitor.

### Inducible neuron differentiation

hESCs were differentiated into cortical neurons following an established protocol (32) with some modifications. When hESCs reached 70–80% confluency, cells were prepared for differentiation. First, any differentiated cells were manually removed, and wells were gently rinsed with sterile PBS. Cells were treated with Accutase and the plate was placed in the incubator for 2 min. The plate was agitated as needed during the incubation time to encourage release of the cells from the plate. After incubating, 1 mL of media was added to cell suspension and cells were singularized by pipetting with a 2-mL serological pipet. Cell suspension was transferred to a 15-mL conical tube and centrifuged at 1200 rpm for 3 min. Media was aspirated from pellet and pellet was resuspended in Induction Media (IM). IM was prepared by supplementing DMEM/F12 with HEPES (Gibco, #11 330 032) with 1X N2 supplement (Gibco, # 17 502 048), 1X MEM Non-essential amino acids, and 1X GlutaMAX (Gibco, #35 050 061). Cells were counted using a hemocytometer and plated for differentiation in IM supplemented with 10 μM ROCK inhibitor and 2 μM doxycycline hydrochloride (Fisher Scientific, BP2653-5). Cells were fed daily with IM supplemented with 2 μM doxycycline hydrochloride for 3 days. On day 4 of differentiation, cells were again singularized with Accutase as above. The cell pellet was resuspended in Cortical Media (CM) supplemented with 10 μM ROCK inhibitor. CM was prepared by mixing equal amounts of DMEM/F12 with HEPES and Neurobasal Medium (Gibco, #21 103 049) and adding 1X B27 supplement (Gibco, #17 504 044), 10 ng/mL BDNF (R&D Systems, 248-BD), 10 ng/mL GDNF (R&D Systems, 212-GD), 10 ng/mL NT3 (PeproTech, 450–03) and 1 μg/mL laminin (Gibco, #23 017 015). Cells were counted using a hemocytometer and plated at 1 million cells per well of a 6-well plate or 7 million cells per 100 mm dish in CM supplemented with 10 μM ROCK inhibitor. Plates and dishes were coated prior to plating with 100 μg/mL poly-D-lysine hydrobromide (Millipore, P0899) and 5 μg/mL laminin (Gibco, #23 017 015). A complete media change with CM was performed the following day. Media was changed every other day until collection on day 11. For sequencing of each cell line, 5–6 biological replicates were used. Each replicate was differentiated in its own dish.

### Immunocytochemistry

hESC-derived neurons were differentiated as above and plated for terminal differentiation on PDL and laminin-coated coverslips. Once neurons reached 11 days post-induction, samples were fixed at room temperature with 4% paraformaldehyde for 10 min. Next, samples were permeabilized using PBS plus 0.5% Triton X 100 (PBS-T) for 5 min at room temperature. Following permeabilization, samples were blocked in 0.1% PBS-T containing 2% bovine serum albumin and 5% normal goat serum for 1 h at room temperature. This blocking buffer was replaced with blocking buffer containing primary antibodies and samples were incubated overnight at 4°C in a humidity chamber. The next day the coverslips were washed three times with 0.1% Triton in PBS for 10 min each. Samples were then incubated in blocking buffer containing secondary antibodies for 1 h at room temperature in the dark. All remaining steps occurred in the dark. The coverslips were washed three times with 0.1% Triton in PBS for 10 min each. Coverslips were mounted with ProLong™ Gold Antifade Mountant with DNA Stain DAPI (Invitrogen, Cat# P36941) and allowed to set for 24 h at room temperature prior to imaging. The following primary antibodies were used: rabbit anti-MAP2 (1:800, Abcam ab32454) and mouse anti-TUBB3 (1:2000, Biolegend #801 201). The following secondary antibodies were used: goat anti-rabbit Cross-Adsorbed Alexa Fluor 488 (1:400; Invitrogen A11008) and goat anti-mouse Cross-Adsorbed Alexa Fluor 594 (1:400; Invitrogen A11005). Images were acquired using a 63X objective on a Zeiss Axio Observer Z1 microscope, keeping exposure time consistent between samples. Representative images were chosen, and image adjustment was performed in ImageJ. In all images, only the color balance was adjusted, and this was performed uniformly across samples. Images were assembled using Adobe Illustrator.

### Cell collection

For hESCs, any differentiated cells were manually removed, and wells were gently rinsed twice with sterile PBS. Sterile filtered 0.5 mM EDTA in PBS was added to wells, and the plate was placed back into the incubator undisturbed for 5.5 min. After incubation, EDTA solution was gently aspirated from wells, being careful to not disturb cells. Using a 2-mL serological pipette, 1 mL of sterile PBS was pipetted down the back of wells to dislodge cells. The cell solution was transferred to a 1.5-mL microcentrifuge tube and centrifuged at 2000 × g for 5 min at 4°C. PBS was aspirated from pellets. Pellets were flash frozen in liquid nitrogen and stored at −80°C until RNA extraction.

For day 11 hESC-derived neurons, media was aspirated from the wells/dish. DMEM/F12 was added to the wells/dish and the cells were scraped to detach them from the plate. The cell suspension was collected in a 15-mL conical tube and spun down at 2000 rpm for 3 min. Media was aspirated from pellet and pellet was resuspended in 1 mL of TRIzol™ (Invitrogen™, #15 596 026). The cell suspension was transferred to a 1.5-mL microcentrifuge tube. The tube was briefly vortexed and incubated at room temperature for 5 min before proceeding with RNA extraction.

### RNA extraction

For hESCs, RNA was harvested using the miRNeasy® Mini Kit (QIAGEN, #1 038 703) following manufacturer's protocol with minor modifications. The work surface, pipettes, and centrifuge rotors were treated with RNAse Away (Life Technologies, #10 328 011) prior to beginning extraction. Pellets were transferred from storage at −80°C to ice. Samples were homogenized in 700 μL QIAzol by pipetting and brief vortexing. Cell lysate was applied to QIAshredder columns (QIAGEN, #1 011 711). Samples were incubated at room temperature for 5 min. Following incubation, 140 μL of chloroform was added to the homogenate and shaken vigorously for 15 s. Samples were incubated at room temperature for 2–3 min and then centrifuged for 15 min at 12 000 × g at 4°C. Approximately 400 μL of the aqueous phase was transferred to a new 1.5-mL microcentrifuge tube. A second chloroform extraction was performed by adding an equal volume of chloroform to the aqueous phase and shaking vigorously for 15 s. The samples were centrifuged for another 15 min at 12 000 × g at 4°C, and the aqueous phase (∼350 μL) was transferred to a new 1.5-mL microcentrifuge tube to which 1.5 volumes of 100% ethanol was added. The contents of the tube were mixed by pipetting and applied to the RNeasy spin column, following manufacturer's instructions for on-column DNase treatment using RNase-Free DNase Set (QIAGEN, #79 254) and the addition of a second wash with Buffer RPE. The optional enrichment step for miRNAs was not performed. For hESC-derived neurons, RNA was harvested using the Direct-zol RNA Miniprep kit (Zymo Research, #R2050) following manufacturer's protocols. For both hESCs and hESC-derived neurons, RNA was eluted in RNase-Free water and stored at -80°C until library construction, for which 1 μg of RNA was used.

### RNA-seq library preparation and sequencing

Total RNA quality for hESC samples and most hESC-derived neuron samples was assessed using the Agilent TapeStation 4200 with RNA ScreenTape Analysis, including RNA ScreenTape (Agilent, #5067–5576), RNA ScreenTape Sample Buffer (Agilent, #5067–5577) and RNA ScreenTape Ladder (Agilent, #5067–5578). All samples measured had an RNA Integrity Number (RIN) of 8.4 or greater.

For hESCs, RNA libraries for RNA-seq were prepared using the NEBNext® Ultra II Directional RNA Library Prep Kit for Illumina® (NEB, #E7760L) following manufacturer's protocol for use with NEBNext Poly(A) mRNA Magnetic Isolation Module (NEB, #E7490). Libraries were checked for quality and average fragment size using ScreenTape analysis, including D1000 ScreenTape (Agilent, #5067–5582) and D1000 Sample Buffer (Agilent, #5067–5602). Concentration of libraries was measured using Qubit™ 2.0 Fluorometer with Qubit™ dsDNA HS Assay Kit (Invitrogen™, #Q32851). Molar concentration of libraries was determined using NEBNext® Library Quant Kit for Illumina® (NEB, #E7630) following manufacturer's protocol for 6 Standards, 100–0.001 pM (Section 3). Quantification of libraries was calculated using the worksheet from NEBioCalculator (v1.15.0, https://nebiocalculator.neb.com). Libraries were diluted to 4nM, pooled, and denatured according to Illumina's protocol. Balancing of pooled libraries was verified by sequencing on the MiSeq, using MiSeq Reagent Cartridge v2 300 cycles (Illumina, #15 033 624) and MiSeq Reagent Nano Kit v2 (Illumina, #15 036 714), at a concentration of 10 pM. Libraries were sequenced by the Center for Genome Innovation at the University of Connecticut Institute for Systems Genomics on the Illumina NovaSeq 6000 at a concentration of 0.7 nM.

For hESC-derived neurons, RNA libraries for RNA-seq were prepared using the TruSeq Stranded mRNA Library Prep Kit (Illumina, #20 020 594) following manufacturer's protocols. Libraries were sequenced on the Illumina NextSeq 550 with settings for dual-index, paired-end sequencing, with 75 cycles per end at a concentration of 1.8 pM.

### RNA-seq data processing

Quality control was performed on RNA-seq reads using FastQC (v.0.11.7) and MultiQC (v.1.10.1) (36). Fastqs were aligned to hg38 using STAR (v.2.7.1a) (37), using options –readFilesCommand zcat –outFilterType BySJout –outFilterMultimapNmax 1 –alignSJoverhangMin 8 –alignSJDBoverhangMin 2 –outFilterMismatchNoverReadLmax 0.04 –alignIntronMin 20 –alignIntronMax 1 000 000 –alignMatesGapMax 1 000 000 –outSAMtype BAM SortedByCoordinate –outWigType bedGraph. GENCODEv25 annotation was used. Equal distribution of reads across the gene body was verified using geneBody_coverage.py (v.3.0.1) from RSeQC (38). Sorted BAM files were used to extract read counts using featureCounts from subread (v.2.0) (39), with option -s 2.

### Differential gene expression (DEG) analysis

DEG analysis was performed in R (v.4.2.1) (40) on extracted read counts using DESeq2 (v.1.36.0) (41). To characterize the neurons we generated via the inducible neuron protocol, we first compared neurons from all genotypes and both backgrounds to WT H9 ESCs. Low gene counts were filtered by removing all genes whose mean of counts across all samples was less than 39, or 1 count per sample. This resulted in a total of 25 440 genes being tested for differential expression. Pairwise differential analysis between WT ESCs and WT neurons, smDEL neurons, and lgDEL neurons was performed using the DESeqDataSetFromMatrix() with design = ∼ Condition_Lineage and a results() contrast of ‘Condition_Lineage’. After comparing inducible neurons to ESCs, we moved on to compare neurons from our genomically edited deletion lines to neurons from their isogenic wild type control. For comparisons of lgDEL neurons to WT neurons and smDEL neurons to WT neurons, unexpressed genes were removed from analysis by removing all genes whose mean of counts across all samples was less than 1. Pairwise differential analysis between WT neurons and lgDEL neurons was performed using the DESeqDataSetFromMatrix() with ‘design = ∼ Condition_Lineage’ and a results() contrast of ‘Condition_Lineage’. This resulted in a total of 34 921 genes being tested for differential expression. Pairwise differential analysis between WT neurons and smDEL neurons was performed using the DESeqDataSetFromMatrix() with ‘design = ∼ Condition_Lineage’ and a results() contrast of ‘Condition_Lineage’. This resulted in a total of 33 019 genes being tested for differential expression. Following this, we performed exploratory PCA analysis to determine the amount of variance in our system. PCA plots were generated using the plotPCA() function from the DESeq2 package on rlog() transformed raw counts for filtered genes. As we noted the genetic background of the cell lines contributes a large amount of variance to the system, we did not attempt to regress the genetic background using model terms in DESeq2. Instead, after analyzing the pairwise comparisons within each background, we used the gene identifier (ENSEMBL ID) to determine which DEGs are reproducible across genetic backgrounds. Following analysis of each genotype independently, we performed additional filtering in DESeq2 to rigorously identify DEGs across both genotypes and backgrounds. To accomplish this, we performed two additional DESeq2 analyses for each lgDEL or smDEL comparisons using DESeqDataSetFromMatrix() designs of ‘∼ Genetic.Background + Condition + Genetic.Background:Condition’ and ‘∼ Condition’ followed by a results() contrast of ‘Condition’. For the lgDEL versus WT neuronal comparison, this resulted in 33 956 genes being tested for differential expression. For the smDEL versus WT neuronal comparison, this resulted in 32 715 genes being tested for differential expression. The results of all three DESeq2 analyses were compared to determine genes shared across all analyses for each lgDEL and smDEL. The resultant shared genes from the lgDEL analyses (691 genes) and smDEL analyses (232 genes), were then compared to each other to determine which DEGs were shared across genotype. Only significant DEGs were used for downstream determination of overlapping, or ‘shared,’ genes. To first obtain significant DEGs, the default DESeq2 FDR setting (alpha) of 0.1 was used and then results tables were subsequently filtered for Benjamini-Hochberg (BH) adjusted p-values (p.adj) of < 0.05. Shared genes were determined using the ggVennDiagram package (v.1.2.2) (42). Results were graphed using either ggVennDiagram, the ComplexUpset package (v.1.3.3) (43), or eulerr (eulerr.co) (44). Permutation tests were conducted to test the significance of these overlaps in the command line. Briefly, lists of all DEGs from both cell lines were shuffled. The same number of overlapping upregulated and downregulated genes were sampled from the top and bottom of the lists, respectively. The lists were joined to determine which genes were overlapping. The number of shared genes was appended to a file. This process was repeated 10 000 times. The number of overlaps was plotted as a histogram and the median of the permuted overlaps was compared to the experimentally obtained number of overlaps to determine the enrichment value. For cases where no permuted value was equal to or greater than the experimentally obtained value, the *P* value was calculated as *p* < 1/(number of permutations). When the permuted value was greater than the experimentally obtained value, the *P* value was calculated as p=(number of values equal to or greater than experimental)/(number of permutations). Boxplots and histograms were generated using ggplot2 (v.3.4.0) (45). To determine gene names from the ENSEMBL ID’s, biomaRt (v.2.52.0) (46,47)) was used with the ENSEMBL archive from April 2018 (host = https://archive.ensembl.org/), the most similar database available for the GENCODEv25 annotation.

### Gene and disease ontology analysis

Shared gene lists generated from DESeq2 analysis for either upregulated genes, downregulated genes, or upregulated and downregulated genes combined were processed for gene (GO) and disease ontology (DO) analysis using the clusterProfiler package (v.4.4.4) (48,49)) and DOSE (v.3.22.1) (50) with functions of enrichGO() and enrichDGN(), respectively with options for pAdjustMethod = ‘BH’ and qvalueCutoff = 0.05. For enrichGO(), the org.Hs.eg.db package (v.3.15.0) was utilized. The universe used was the list of genes in each DESeq2 object generated via the ‘Background_Condition’ contrasts. GO results were simplified using the simplify() function from clusterProfiler with options of cutoff = 0.7 or 0.8. Dotplots were generated using ggplot2 on GO and DO results ordered first by the adjusted *P*-value then by foldEnrichment. The foldEnrichment score was calculated by dividing the *GeneRatio* by the *BgRatio* values for each result. A Gene-Concept Network plot was generated using the enrichplot package (v.1.16.2) using a foldChange object made with dplyr package (v.1.0.10). For Figure 4A, the disgenet2r package (v.0.99.3) (51) was used, with a default universe of all genes.

### snoGloBe prediction and analysis

SnoGloBe was used to predict interactions of *SNORD116* C/D box snoRNAs with the 42 genes shared between small and large deletion models across both genetic backgrounds, as described previously (33). Per the authors’ recommendation to narrow the number of predictions obtained, we selectively kept the predicted interactions having at least three consecutive windows with a score of greater than or equal to 0.98 for further analysis, using options -t 0.98 -m -w 3. For the control analysis of 100 lists of 42 genes, we generated lists of genes which did not differ significantly from our list of 42 dysregulated genes (via the Wilcoxon test) in length, GC content, or expression in our inducible neuron system. These lists were then analyzed using snoGloBe for predicted binding of *SNORD116* using the same settings as above. For plotting the distribution of the predicted region of interaction of snoRNAs, the center of each binding event was determined and then the relative position of the binding event was calculated. The relative position of C/C’ and D/D’ boxes was calculated and then plotted using the grid.rect() function of the grid package (v.4.2.1). Genomic feature coverage was determined using bedtools (v.2.29.0) for hg38. Only transcripts with support levels of 1–3 and a tag of basic were used.

### Western blot

hESCs were differentiated into neurons as described above. Once neurons reached 11 days of maturity, samples were collected as described above. Upon thawing, cell pellets were lysed in 100 μL of cell lysis buffer (Cell Signaling Technology, Cat# 9803) containing protease inhibitors (1 mM phenylmethylsulfonyl fluoride [PMSF] and 1:200 dilution of Protease Inhibitor Cocktail Set III [Calbiochem, Cat# 539 134]). Total protein concentration was measured using a BCA assay. For each sample, 10 μg of protein in 4X Laemmli Sample Buffer (Bio-Rad, Cat# 1 610 747) was separated by SDS-PAGE using 4–20% Mini-PROTEAN® TGX™ Precast Protein Gel (Bio-Rad, Cat# 4 568 093). Protein was transferred to PVDF membrane using the TransBlot Turbo system (Bio-Rad). Membrane was blocked using 5% w/v non-fat milk in 1X TBS (Bio-Rad, Cat# 1 706 435) plus 0.05% Tween-20 (TBS-T) at room temperature for 1 h on orbital shaker set to 80 rpm. Membrane was then incubated in blocking buffer containing primary FGF13 antibody overnight at 4°C while rocking. Membrane was washed with TBS-T at room temperature, incubated in blocking buffer containing secondary antibody for 1 h at room temperature on orbital shaker set to 80 rpm, and then washed again in TBS-T. All washes were done three times for 10 min each at room temperature on orbital shaker set to 80 rpm. Membrane was imaged using Clarity Western ECL substrate (Bio-Rad, #1 705 060) on the ChemiDoc Touch imaging system (Bio-Rad) using rapid auto exposure setting. Following the imaging of FGF13, the blot was washed a few times quickly with TBS-T to remove the ECL substrate and then put in fresh TBS-T on the orbital shaker set to 80 rpm for 5 min. The blot was stripped using Restore™ PLUS Western Blot Stripping Buffer for 15 min at 37°C while rocking. The blot was then blocked again using 5% w/v non-fat milk in TBS-T and the staining process was repeated for loading control GAPDH. The following antibodies were used: rabbit anti-FGF13 (1:600; ThermoFisher 26235–1-AP), mouse anti-GAPDH (1:10 000; Millipore-Sigma MAB374), anti-rabbit IgG HRP-linked (1:2000; Cell Signaling #7074) and anti-mouse IgG HRP-linked (1:2000; Cell Signaling #7076). FGF13 decrease was quantified as previously described (52) using ImageJ software.

## Results

### Isogenic cell line pairs utilizing an inducible neuron system were generated to evaluate the effects of SNORD116 loss in the context of PWS

**Figure 1.**
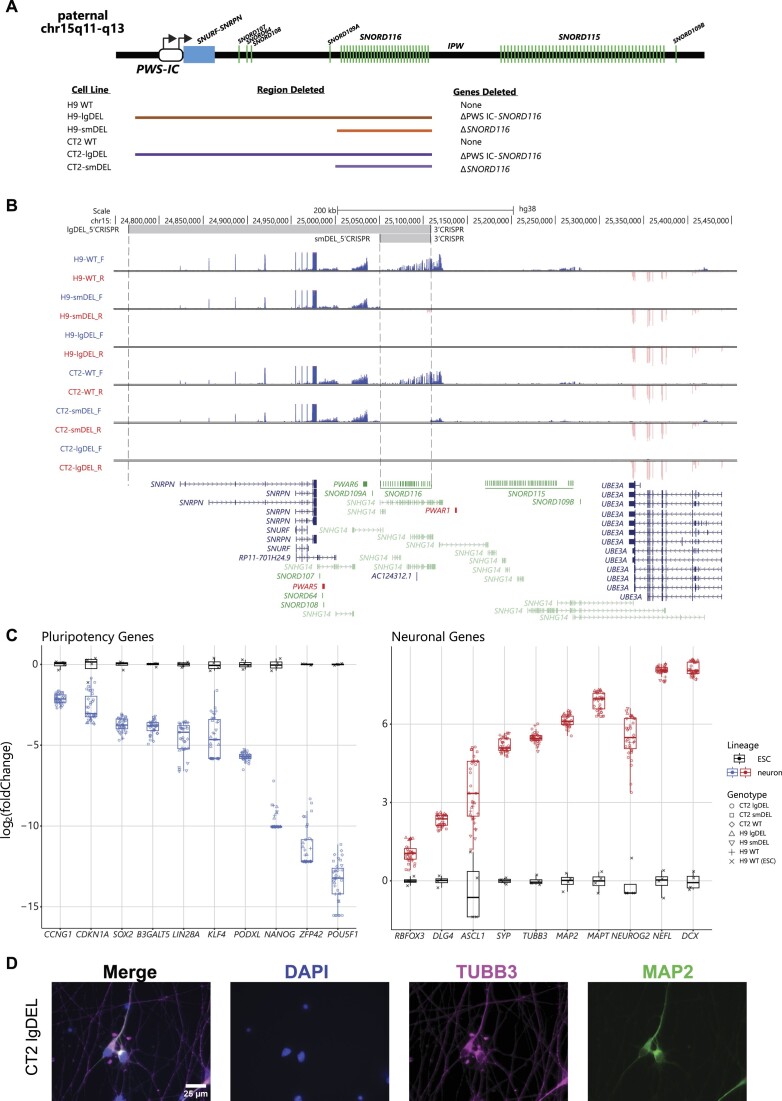
Summary of genomic editing and characterization of differentiated neurons. (**A**) A schematic of the deletions present in our model system. Not drawn to scale. (**B**) UCSC Browser image of the chromosome 15q11-q13 locus displaying representative bigwig tracks from each genetic background and genotype. Blue tracks show RNA signal from the sense strand; red tracks show RNA signal from the antisense strand. Top track shows CRISPR gRNA binding locations; gray shading indicates deleted region. GENCODEv25 gene annotations are shown at the bottom; protein-coding genes are shown in blue, noncoding genes are shown in green, and To Be Experimentally confirmed (TEC) biotype genes are shown in red. Some isoforms have been removed for clarity. (**C**) Box and whisker plots displaying a subset of significant DEGs (p.adjust < 0.05) in all cell lines across both genetic backgrounds as inducible neurons compared to wild type H9 ESCs (*n* = 4–6 biological replicates). ESC samples are shown in black and neuron samples are shown in blue or red. Genotype is represented by data point shape. The y-axis represents log_2_(foldChange) and the x-axis displays individual gene names for either pluripotency markers (*left*) or neuronal markers (*right*). Pseudocount was added to counts of all genes prior to calculation of log_2_(foldChange). (**D**) Representative immunofluorescent imaging of CT2 lgDEL cell line for canonical neuronal proteins TUBB3 and MAP2 in addition to nuclear marker DAPI at day 11 post-induction. Images were acquired at 63x magnification. Scale bar represents 25 μm.

We initially set out to identify genes that might be consistently dysregulated in PWS. Several studies have reported DEGs between postmortem brain tissue and iPSC-derived neurons from PWS patients and controls. However, when we analyzed these differential gene expression data, few genes were consistently dysregulated in the disease context (Supplemental Figure S1) (28,30)). Further, the genes that were shared between these studies do not show clear connections to PWS-related phenotypes through gene ontology analysis (Supplemental Figure S1). We reasoned that one major contributor to this lack of concordance could be due to differences in genetic backgrounds between PWS patients and controls, as genetic background in cellular models has been well documented in literature to play a significant role in differential gene expression (53–57). To generate models of PWS that could be directly compared to isogenic controls, we engineered two different deletions on the paternal chromosome 15q allele in two distinct hESC lines by utilizing CRISPR/Cas9 editing with guide RNAs (gRNAs) designed to target up- and downstream of our regions of interest (Methods) (Supplemental Table S1, Supplemental Figure S2). One deletion spanned from alternative promoters of the *SNRPN* transcript upstream of the PWS-IC to the distal end of the *SNORD116* snoRNA cluster (termed ‘lgDEL’ model) (Figure 1A). The other deletion encompassed just the *SNORD116* cluster (termed ‘smDEL’ model) (Figure 1A). All six cell lines (H9 WT, H9-smDEL, H9-lgDEL, CT2 WT, CT2-smDEL and CT2-lgDEL) were further engineered to contain a stably integrated cassette allowing for rapid induction of neurons using human NGN2 (32) (Materials and methods) (Supplemental Figure S2). Analysis of top off-target sites of CRIPSR gRNAs (Materials and methods) showed no evidence of editing (Supplemental Figure S3). Additionally, a CytoSNP array analysis revealed no significant copy number changes compared to unedited lines (Supplemental Figure S4). Neurons generated using the NGN2 induction approach did not have any noticeable phenotypic differences between any of the deletions and controls in either background (Supplemental Figure S5). Examination of RNA-Seq signals in neurons generated by the inducible neuron system at the PWS locus confirmed the size of each deletion and targeting of the paternal allele due to lack of expression from the deleted region (Figure 1B). Comparing all inducible neurons to WT H9 ESCs showed that canonical pluripotency genes were downregulated, and canonical neuronal genes were upregulated across all lines (Figure 1C). Systematic analysis of gene expression between neurons and wild type hESCs revealed largely the same DEGs and gene ontology analysis of the shared DEGs upregulated in neurons showed enrichment of terms of neuron-related processes, components and function (Supplemental Figure S5). Inducible neurons also robustly express neuronal proteins TUBB3 and MAP2 by day 11 post-induction (Figure 1D, Supplemental Figure S5).

### Eliminating expression from *SNHG14* promoters results in expression changes consistent with PWS phenotypes

**Figure 2.**
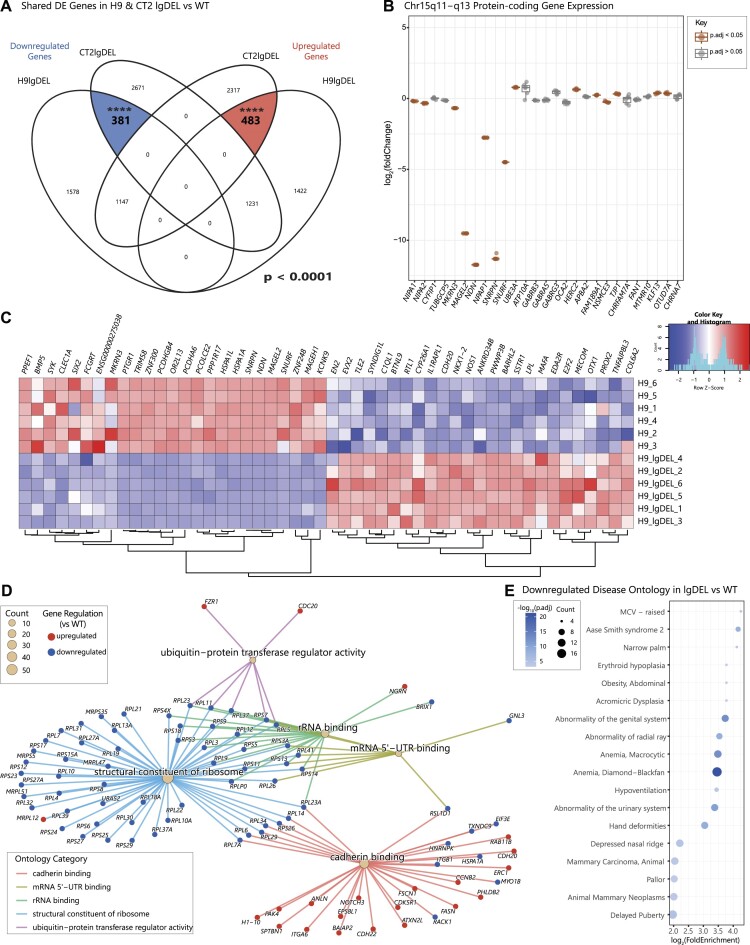
Analysis of gene set perturbed in lgDEL cell lines versus controls as neurons. (**A**) Venn diagram displaying overlap of significant DEGs (p.adjust < 0.05) for lgDEL lines in both genetic backgrounds versus their isogenic WT controls. Left side of diagram represents significant downregulated (log_2_FoldChange < 0) DEGs with shared genes highlighted in blue; right side of diagram represents significant upregulated (log_2_FoldChange > 0) DEGs with shared genes highlighted in red. Significance of overlaps (*P* < 0.0001) determined via a permutation test. (**B**) Box and whisker plot showing expression of a subset of protein-coding genes in the chromosome 15q11-q13 region. Pseudocount was added to counts of all genes prior to calculation of log_2_(foldChange). Significant DEGs (p.adjust < 0.05) are shown in orange. (**C**) Heatmap showing 50 most dysregulated significant DEGs in lgDEL vs WT H9 genetic background. Top 25 up- and downregulated genes were determined by average log_2_(foldChange) between CT2 and H9 backgrounds. Shading indicates row z-score, with blue denoting downregulated gene expression and red denoting upregulated gene expression. Rows represent samples; columns represent individual genes. (**D**) Gene-concept network plot displaying GO terms of the molecular function (MF) category for all shared dysregulated genes. Main nodes (tan) correspond to the MF category with colored lines connecting to nodes of genes found in each category. Size of the main node corresponds to the number of DEGs in our data set contained within each ontology term. Colors of the gene nodes correspond to the log_2_(fold Change) for each DEG in lgDEL vs WT control; red indicates log_2_(foldChange) > 0, blue indicates log_2_(foldChange) < 0. (**E**) Dot plot displaying disease ontology results for shared downregulated genes. The x-axis represents the log_2_ fold enrichment value, and y-axis shows disease ontology terms. Size of the dot corresponds to the number of DEGs in our data set contained within each ontology term. Shading of the dot corresponds to the negative log_10_ of the adjusted *P*-value.

Having confirmed that each of the lines harbored the desired genomic edits and generated neurons reliably, we set out to compare gene expression patterns across neurons. DEG analysis (Methods) (Supplemental Figure S6) of the lgDEL model neurons compared to WT neurons identified 483 upregulated DEGs and 381 downregulated DEGs shared across genetic backgrounds (Figure 2A) (Supplemental Table S2). This was a ∼5-fold and ∼3-fold enrichment of shared DEGs based on random permutations of similarly sized gene lists, respectively (*P* < 0.0001) (Supplemental Figure S6). When we inspected the PWS locus specifically, genes in the deletion were significantly differentially expressed as expected. However, we also noticed genes outside the boundaries of the engineered deletion were differentially expressed (Figure 2B, Supplemental Figure S6). We examined the most dysregulated genes, which were consistent across both backgrounds (Figure 2C, Supplemental Figure S6). Gene ontology analysis (Materials and methods) on all 864 dysregulated genes revealed Molecular Function category terms related to ribosome structure, rRNA binding and mRNA 5′-UTR binding, among others (Figure 2D, Supplemental Table S3). Interestingly, the DEGs present in the Structural Constituent of Ribosome category seem to be enriched for genes with lower expression in the brain compared to other tissue types (Supplemental Figure S7) (58). While disease ontology analysis on the 483 shared upregulated DEGs only returned two significant terms (Supplemental Figure S7), analysis of the 381 shared downregulated DEGs resulted in ontology terms related to phenotypes seen in PWS patients, such as delayed puberty, abnormality of the genital system and obesity (Figure 2E, Supplemental Table S4). These results support the relevance of the lgDEL model in studying PWS.

### Deletion of *SNORD116* alone is necessary to determine the targets and functions of *SNORD116* snoRNAs

**Figure 3.**
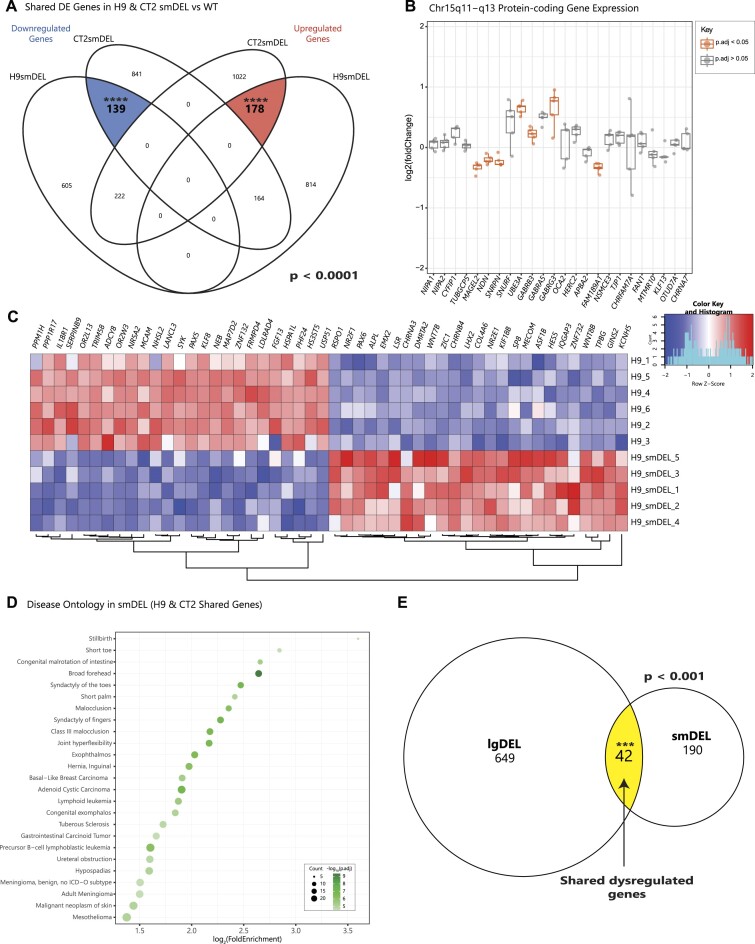
Analysis of gene set perturbed in smDEL versus controls as neurons. (**A**) Venn diagram displaying overlap of significant DEGs (p.adjust < 0.05) for smDEL lines in both genetic backgrounds versus their isogenic WT controls. Left side of diagram represents significant downregulated (log_2_FoldChange < 0) DEGs with shared genes highlighted in blue; right side of diagram represents significant shared upregulated (log_2_FoldChange > 0) DEGs with shared genes highlighted in red. Significance of overlaps (*P* < 0.0001) determined via a permutation test. (**B**) Box and whisker plot showing expression of a subset of protein-coding genes in the chromosome 15q11-q13 region. Pseudocount was added to counts of all genes prior to calculation of log_2_(foldChange). Significant DEGs (p.adjust < 0.05) are shown in orange. (**C**) Heatmap showing 50 most dysregulated significant DEGs in smDEL versus WT H9 genetic background. Top 25 up- and downregulated genes were determined by average log_2_(foldChange) between CT2 and H9 backgrounds. Shading indicates row z-score, with blue denoting downregulated gene expression and red denoting upregulated gene expression. Rows represent samples; columns represent individual genes. (**D**) Dot plot displaying disease ontology results for all shared dysregulated genes in smDEL lines across both backgrounds. The x-axis represents the log_2_ fold enrichment value, and y-axis shows top 25 disease ontology terms. Size of the dot corresponds to the number of DEGs in our data set contained within each ontology term. Shading of the dot corresponds to the negative log_10_ of the adjusted *P*-value. (**E**) Venn diagram displaying overlap of all significant DEGs (p.adjust < 0.05) after additional filtering for both genetic backgrounds and genotypes versus isogenic WT controls. Yellow shading denotes significant shared dysregulated gene set. Significance of overlap (*p* < 0.001) determined via a permutation test.

While a large deletion model is relevant to many PWS cases, a recent report of a microdeletion encompassing the *SNORD116* cluster suggests these genes may be the primary contributor to the PWS phenotype (21). Therefore, we made a targeted deletion of the *SNORD116* C/D box snoRNA cluster (smDEL) that leaves promoters of the *SNHG14* parent transcript intact and retains expression of *SNURF*-*SNRPN*. DEG analysis performed in a similar fashion as above (Methods) (Supplemental Figure S8) (Supplemental Table S5) revealed 178 upregulated DEGs and 139 downregulated DEGs shared across genetic backgrounds of our smDEL models (Figure 3A), a ∼7-fold and ∼9-fold enrichment of shared DEGs versus random permutations respectively (*P* < 0.0001) (Supplemental Figure S8). Similarly to the lgDEL model, the smDEL also impacted gene expression in the PWS locus beyond the bounds of the deletion (Figure 3B, Supplemental Figure S8). Also similarly to the lgDEL model, the most dysregulated genes were consistent across genetic backgrounds. However, the reduced number of genes did result in fewer relevant gene ontology categories (Supplemental Table S6). Surprisingly, this reduced set was enriched for disease ontology terms related to phenotypes seen in PWS patients, like short toe and short palm (Figure 3D, Supplemental Table S7) (2). As there is a mouse model with a *Snord116* deletion, we decided to compare our smDEL DEGs to the DEGs described in an RNA-Seq analysis of this mouse model versus WT littermates (59). We observed a subset of shared DEGs between these two models (Supplemental Table S8), corresponding to a modest enrichment when tested via a permutation test (*P* = 0.022). The 116 shared DEGs between the two models is disregarding directionality. For concordant expression (i.e. a gene is upregulated in human smDEL vs WT and also upregulated in mouse *Snord116* deletion vs WT), the number of shared DEGs reduced by half, resulting in only 58 shared genes. We hypothesized that the differences between human and mouse may be driven by differential regulation of the PWS locus across species. Techniques have been developed combining chromatin immunoprecipitation followed by sequencing (ChIP-seq) and machine learning approaches to predict states of regulation at the chromatin level (60). This tool, chromHMM, uses a combinatorial code of the presence or absence of histone modifications to predict states of activity throughout the genome for various timepoints and tissue types. When examining these predicted states in human and mouse brain tissue (61,62)) at the PWS locus, very little shared predicted states can be observed (Supplemental Figure S9). This is in contrast to other nearby syntenic loci that have conserved patterns of chromatin state predictions in the brains of mice and humans (Supplemental Figure S9) This finding, in addition to the well-established gene expression differences across the two species, suggests that human and mouse regulate this region quite differently.

### Comparison of small and large deletion models reveals a novel and robust regulatory network of genes consistently dysregulated in PWS-like systems

Having demonstrated that DEGs in each set of models identified genes enriched for PWS relevant phenotypes, we wondered if any DEGs might be shared across lgDEL and smDEL models. We hypothesized that genes shared across all comparisons are central to the disorder and therefore important to focus on. We further filtered our DEGs from the lgDEL and smDEL models (Methods) (Supplemental Figure S10) (Supplemental Tables S9–S12) which resulted in 691 total DEGs in the lgDEL model and 232 total DEGs in the smDEL model. After overlapping these two lists, we found 42 genes shared between both genetic backgrounds and deletions (Figure 3E), a ∼3-fold enrichment of shared DEGs versus random permutations (*P* < 0.001) (Supplemental Figure S10). The list of 42 genes contains 8 transcription factors (TFs) and 3 genes located within the PWS locus at chr15q11-q13 (Table 1 and Supplemental Table S13).

**Table 1.** List of 42 consistently dysregulated genes across genetic backgrounds and genotypes

*ABLIM1*	*DUSP4*	*IL1RAPL1*	*NEB*	*PLPP3*	*RSL1D1*
*ADGRB1*	*EFNB1*	*IRX5*	*NR5A2*	*PPP1R17*	*SNORD116-20*
*AMH*	ENSG00000250284	*KCTD10*	*PAX5*	*PTN*	*SNHG14*
*CDH20*	*FGF13*	*KIF24*	*PAX6*	*PTX3*	*SOX21*
*CDK5R1*	*GRM7*	*LUZP2*	*PIDD1*	*RAB7A*	*SULF2*
*COL18A1*	*HSPA1L*	*MAGEL2*	*PLAGL1*	*RECQL4*	*SYK*
*DPP6*	*IL18R1*	*MYBL2*	*PLK2*	*RIMKLA*	*ZIC2*

**Figure 4.**
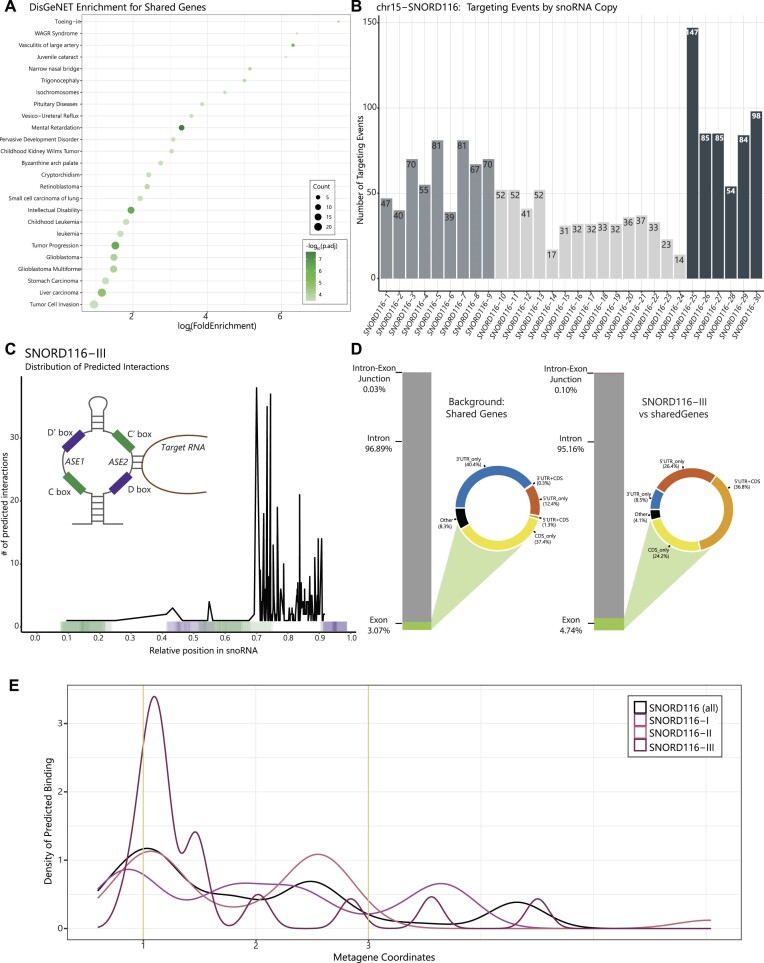
Interrogation of shared dysregulated genes and analysis of *SNORD116* predicted targets. (**A**) Dot plot displaying DisGeNET results for 42 shared dysregulated genes. The x-axis represents the log_2_ fold enrichment value, and y-axis shows top 25 ontology terms. Size of the dot corresponds to the number of DEGs in our data set contained within each ontology term. Shading of the dot corresponds to the negative log_10_ of the adjusted *P*-value. Note Tumor Cell Invasion, Liver carcinoma, Tumor Progression ontologies contain *SNHG14* as one of the DEGs driving these categories. (**B**) Bar plot displaying the number of predicted targeting events per copy of *SNORD116*. Colors of the bars correspond to the three subgroups of *SNORD116*: *SNORD116*-I (copies 1–9), *SNORD116*-II (copies 10–24) and *SNORD116*-III (copies 25–30). (**C**) Plot displaying distribution of prediction interactions for *SNORD116*-III. The x-axis corresponds to the relative position within snoRNA copies, and y-axis represents the number of predicted interactions for which the center of the predicted binding interaction was used (black line). Color-coded bar on the x-axis indicates the position of C/C’ and D/D’ boxes found in snoRNA copies, indicated by green and purple, respectively. (**D**) Bar charts representing the proportion of exon, intron, and intron-exon junctions in the shared 42 dysregulated genes (Background: Shared Genes) and the predicted targeting of *SNORD116*-III copies on those shared genes (*SNORD116*-III vs Shared Genes). Exon category is subdivided based on genic location and displayed as donut plots. Coloring of donut plots is based on exon category; 5′UTRs are represented in orange, 3′UTRs are represented in blue, CDS is represented in yellow, and any portion of exonic sequence not falling under those categories is termed ‘other’ and shown in black. (**E**) Metagene plot of predicted binding sites for *SNORD116* versus the shared dysregulated gene set. Black line shows average of all *SNORD116* groups. Various pink lines show each individual group (I-III). Metagene coordinates (x-axis) of 0–1 represent the 5′UTR, coordinates 1–2 represent the gene body, and coordinates 2 + represent the 3′UTR. The density of the predicted binding is on the y-axis.

*SNHG14* is one of the genes included in this list. While this gene is unexpressed in lgDEL models, it is still expressed in smDEL models. *SNORD116-20* is also included, though it should be noted that this transcript is deleted in both cell lines and thus not expected to be expressed. While binding profiles of the TFs contained within this list have not been studied specifically in the context of PWS, we turned to the Enrichr gene set enrichment database that has compiled many different resources of experimental and predicted DNA binding and protein-protein interactions (63,64)). Specifically, we queried the Enrichr_Submissions_TF-Gene_Cooccurrence library, which has been compiled from over 300 000 gene set submissions, to evaluate the co-occurrence of our shared genes and TFs. This approach has proven effective in both identifying established gene interactions and uncovering new ones (65). When we analyzed the set of 42 shared genes, we found that 6 out of the 8 TFs in the shared gene list showed significant co-occurrence (Supplemental Table S14). Further, disease ontology analysis on the 42 shared genes (Methods) revealed among the most significant ontology categories were those associated with Intellectual Disability/Mental Retardation (Figure 4A) (Supplemental Table S15), a trait commonly associated with PWS (2). Though we analyzed gene expression in a neuronal model, many of the disease ontology enrichments we obtained are not directly related to neuronal function. When we examined expression of the 42 shared genes across dozens of tissues profiled by the Genotype-Tissue Expression (GTEx) project (66), we noticed many of these genes were expressed across multiple tissue types, not just the brain, suggesting they might be co-expressed in different contexts (Supplemental Figure S11). In addition, these genes had significantly lower median LOEUF score, a measure of a gene's likelihood to have a deleterious mutation in the healthy population, compared to the remainder of the genes contained within the gnomAD database (v.2.1.1, https://gnomad.broadinstitute.org/) (67) (Supplemental Figure S11), further supporting the potential disease relevance of this gene network.

### *SNORD116* snoRNAs are predicted to directly regulate a subset of our novel gene network

We next wondered whether any of these genes may be directly regulated by *SNORD116* snoRNAs. We employed a novel C/D box snoRNA prediction tool, snoGloBe (33), which predicted a significant enrichment of *SNORD116* interactions with our shared gene list versus several control analyses (Methods). Examining the distribution of these predicted targeting events revealed that 35 of the 42 genes are predicted to be targeted by *SNORD116* (Supplemental Figure S12) (Supplemental Table S16). When we plotted the number of predicted binding events per copy of *SNORD116*, we observed a correlation between the number of predicted binding events and the established breakdown of *SNORD116* snoRNAs into its three subgroups: *SNORD116*-I (*SNORD116-1* to *SNORD116-9*), *SNORD116*-II (*SNORD116-10* to *SNORD116-24*) and *SNORD116*-III (*SNORD116-25* to *SNORD116-30*) (12,22)) (Figure 4B). Interestingly, we noted that *SNORD116*-III copies showed the highest number of predicted binding events per copy.

**Figure 5.**
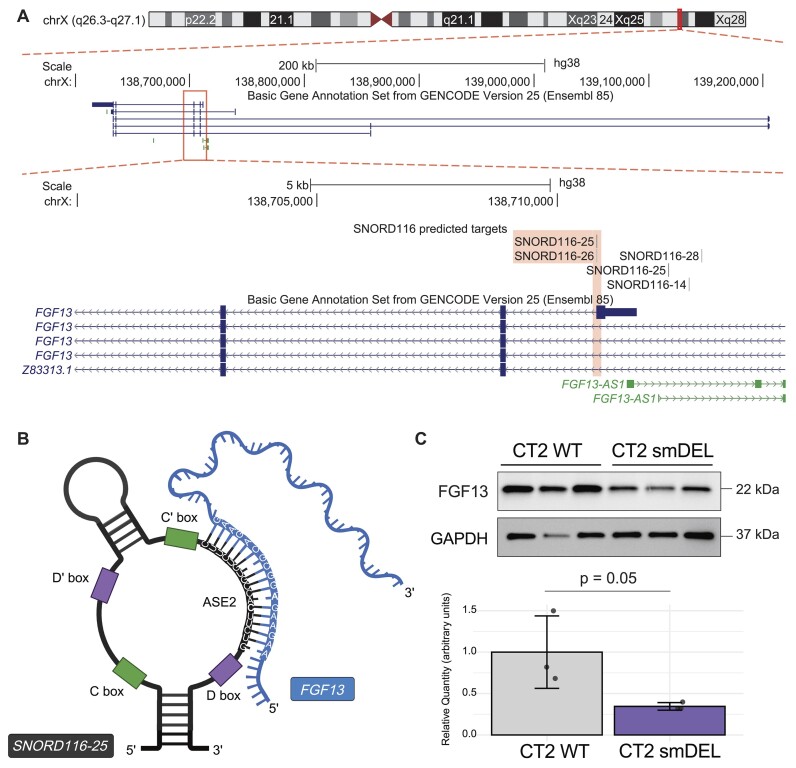
Investigation of *FGF13*, a predicted *SNORD116* target. (**A**) UCSC Browser image of the *FGF13* locus displaying BEDtracks of *SNORD116* predicted binding. Chromosome ideogram indicates location of *FGF13* on X chromosome in red. Top track shows all *FGF13* gene isoforms in GENCODEv25 annotation. Bottom track shows zoomed in view, with predicted binding shown to occur in 5′ exonic region of one transcript of *FGF13* (ENST00000315930.10). (**B**) Cartoon depicting one predicted RNA-RNA interactions between *SNORD116-25* and *FGF13*. Created in BioRender. Gilmore, R. (2024) BioRender.com/k76r157. Not drawn to scale. (**C**) Western blot image of FGF13 and GAPDH in CT2 WT and CT2 smDEL inducible neurons harvested at day 11 post-induction (*n* = 3 biological replicates each). Barplot shows quantification of FGF13 decrease. Significance (*P* = 0.05) determined by the Wilcoxon Rank Sum test.

To analyze the significance of our results, we first compared the number of predicted targeting events per snoRNA copy of *SNORD116* versus our shared dysregulated genes to *SNORD115* versus our shared dysregulated genes (Supplemental Figure S12) (Supplemental Table S17). We saw that *SNORD116* copies have an enrichment of predicted targeting events per copy versus shared dysregulated genes compared to *SNORD115* versus the same gene set. Additionally, genes with predicted targeting events were significantly enriched for predicted targeting by *SNORD116-III* compared to *SNORD115* copies (Supplemental Figure S13). Another control we performed was a permutation test of *SNORD116* versus 100 individual lists of 42 genes, which did not differ significantly from our dysregulated gene list in length, GC content, or expression in our inducible neuron system. We observed a significant ∼2.5-fold enrichment of the mean, median, and sum of *SNORD116-III* predicted targeting events on the shared dysregulated gene list compared to these randomly permuted lists (*P* < 0.01) (Supplemental Figure S14). We also wanted to examine which part of the snoRNA copies were predicted to interact with the shared dysregulated genes. When we plotted the distribution of predicted binding events across snoRNA copies (Materials and methods), we observed predicted binding events for *SNORD116*-III copies mainly occur upstream of the D box at the second antisense element (ASE2) (Figure 4C), a portion of this class of snoRNA that typically interacts with target RNAs (68,69)). This trend is less clear for other *SNORD116* groups and for our control *SNORD115* copies versus shared dysregulated genes, which show a greater portion of predicted targeting events occuring in the C/C’ boxes (Supplemental Figure S15). Having determined the portion of the snoRNA predicted to interact with the target genes, we wanted to examine where in the shared dysregulated genes the snoRNA copies were predicted to bind. Similarly to the findings presented by Deschamps-Francoeur et al. (33), when we compared the background genomic feature coverage of our shared genes list to the genomic feature coverage of *SNORD116-III* predicted binding events, we saw an enrichment in both exon and intron-exon junction categories. Most notably, there was a large increase in coverage of 5′-UTR and 5′-UTR + CDS regions for *SNORD116*-III predicted binding events versus shared dysregulated genes (Figure 4D–E). We determined this enrichment by comparing it to the space of 5′-UTR and 5′UTR + CDS present in our shared dysregulated gene set, termed ‘Background: Shared Genes’ and to the space of 5′-UTR and 5′-UTR + CDS covered by the predicted binding of other SNORD116 groups versus the shared dysregulated gene set (Supplemental Figure S16). Seeing such a dramatic increase in predicted binding events in this region compared to our controls may suggest a role for *SNORD116*-III in regulation of translation of the shared dysregulated genes. One of the genes we found particularly intriguing was *FGF13*, which was downregulated in all our PWS-like models. Upon examination of the *SNORD116*-III predicted targeting of this gene, we noticed that there are predicted targeting events within the first exon of one of the isoforms of this gene (Figure 5A, B). To examine if these consistent significant differences at the transcriptional level correlated with differences at the translational level, we performed a western blot for FGF13. Indeed, we observed a significant decrease in FGF13 protein in the smDEL samples versus the isogenic wild type control (*n* = 3) (Figure 5C, Supplemental Figure S17) (Supplemental Table S18). Whether this is a direct effect of *SNORD116* loss or a downstream consequence will require extensive further experimentation.

## Discussion

While it has long been understood that perturbations of the chr15q11-13 region cause PWS, it is unclear if the genes included in the deletions are directly related to PWS phenotypes, if genes regulated by them are to blame, or if it is some combination of these effects. Multiple studies have attempted to address this issue by characterizing gene expression in postmortem PWS brain tissues and neurons differentiated from PWS patient-derived pluripotent stem cell lines to identify genes dysregulated in this disorder (27–31). While these studies indicate gene expression is indeed dysregulated in PWS patient samples, our analysis here showed few genes had consistent dysregulation across a subset of these studies (Supplemental Figure S1A). Furthermore, the genes that showed consistent trends across these studies seemed to have limited relevance to PWS based on gene ontologies (Supplemental Figure S1B). This discordance in gene expression patterns could be attributed to multiple reasons, both technical and biological. Obtaining controls from otherwise healthy donors for postmortem brain tissue comparisons matched for age, sex, genetic background and postmortem delay is extremely challenging. For iPSC-based experiments, the background of genetic variants outside of the chr15q11-13 region could be substantially different between PWS patients and otherwise healthy controls. This is problematic as multiple studies have established that genetic background of induced pluripotent stem cells (iPSCs) can contribute substantially to changes in gene expression (53–57). Even heterogeneity found in neuronal differentiation of these cellular models can prove to be a challenge in generating reproducible differential gene expression results (70). These background effects could be potentially mitigated in PWS patient derived cells if the missing genetic material could be restored. However, the size of the deletions frequently present in PWS patients poses a challenge for replacing missing genetic information to generate such isogenic controls.

To combat these issues, we utilized multiple isogenic cell lines and an inducible differentiation protocol to generate reproducible, homogenous neurons. The caveats of this system include a lack of electrically active neurons, a more artificial path through neuronal differentiation, and that these are cortical neurons, as opposed to hypothalamic neurons which are most often implicated in PWS physiology (71). The lgDEL model harbors a deletion encompassing all promoters of the *SNRPN* transcript, which eliminates transcription of the host gene and, therefore, processing and expression of *SNORD116*. The smDEL model harbors a targeted deletion of just the *SNORD116* snoRNAs, designed to model the smallest known deletion to still result in PWS phenotypes (21). As *SNORD116* snoRNAs are not polyadenylated and thus not enriched for during polyA-RNA-Seq, most *SNORD116* copies do not meet cutoffs to be called DEGs in our data set. However, the lack of signal from the *SNORD116* locus demonstrated successful deletion of the region in both models (Figure 1B).

Notably, in the lgDEL model we saw differential expression of a subset of ribosomal protein genes. While these genes are typically thought to be utilized similarly across most tissues, the set of ribosomal DEGs identified here have generally lower expression in brain compared to other tissues profiled by GTEx (Supplemental Figure S7A). This could suggest that due to their lower starting expression levels, these proteins are more sensitive to small perturbations. As neither *SNURF* nor *SNRPN* are significantly dysregulated in the smDEL model (Figure 3B, Supplemental Figure S8B), this analysis may demonstrate separable functions of *SNURF-SNRPN* and *SNORD116* snoRNAs. Specifically, *SNURF* and/or *SNRPN* may have a specialized role in ribosomal gene expression while the *SNORD116* snoRNAs may have a completely different role. C/D box snoRNAs have generally been shown to bind and modify ribosomal RNAs (25,72)). However, both *SNORD116* and *SNORD115* snoRNA gene clusters in the chr15q11-13 region are known as orphan snoRNAs and do not show any sequence homology with rRNAs. Previous studies have predicted binding events of these snoRNAs using basic sequence matching approaches, however these results have not been confirmed in a disease-relevant context (26,73,74). Upon further investigation, none of the genes previously predicted to be targeted by *SNORD116* (26) were consistently differentially expressed in our smDEL model across both genetic backgrounds. More recent snoRNA prediction tools have employed machine learning techniques trained on large scale RNA-RNA interaction data to develop models for systematic prediction of such interactions (33). Application of this tool to the consistently dysregulated gene set revealed increased numbers of predicted targeting events by *SNORD116*, particularly amongst group III copies. Importantly we leveraged *SNORD115* copies as controls in this analysis. As *SNORD115* is also a cluster of C/D box snoRNAs contained within the same locus and its deletion alone has no observable phenotypes (75), it serves as a relevant comparator. The predicted *SNORD116* binding sites were facilitated primarily by the second antisense element (ASE2) of *SNORD116*-III sequences (Figure 4C), consistent with described mechanisms of C/D box snoRNA targeting (68,69)). The predicted binding sites on the consistently dysregulated genes were particularly enriched at 5′-UTR and 5′-UTR + CDS regions (Figure 4D-E) suggesting a potential role for *SNORD116*-III copies in modulating transcript stability and/or translation (76). Even though we observed a slight enrichment of predicted binding at intron-exon junctions (Figure 4D) and snoRNAs have been implicated in alternative splicing (77,78)), we do not believe this small enrichment suggests a significant role for *SNORD116* in splicing. Additionally, our analysis suggests that even amongst *SNORD116* there is bias in gene regulation (Figure 4B). The *SNORD116*-III copies have been proposed to have arisen relatively recently on the primate lineage (26). With the advent of expanded genome sequencing and assembly of hundreds of additional mammalian species (79,80)) a more complete picture of the evolution of this locus can be obtained. These multi-species alignments actually suggest presence of all 30 *SNORD116* copies in most placental mammal species. They also indicate instead of recent gain of *SNORD116*-III copies on the primate lineage, loss of this group on a sublineage of glires that includes mice and rats as well as a sublineage of laurasiatherians including many bats. These specific lineage losses, along with vastly different regulation within the PWS locus across species (Supplemental Figure S9A) could begin to explain lack of PWS relevant phenotypes (e.g. hyperphagia and obesity) observed in mouse models of PWS. Subsequent targeted deletions of individual *SNORD116* groups could shed more light on these findings. Additionally, it is quite possible that other mammals could be better models of this disorder. While we have endeavored to create a well-controlled experimental design at the genetic level, there are several limitations of this study. We are unable to differentiate between the effects of loss of *SNORD116* expression and loss of the genetic region itself. As mentioned above, the SNORD116 DNA sequences may play a role in silencing of the locus. Furthermore, other work from our group indicated regions such as *IPW* can form long range interactions to *MAGEL2* and other surrounding genes (81). Thus, the deletions we have constructed, even the smallest one, could have large scale impacts on chromatin organization and result in *MAGEL2* dysregulation. More targeted deletions that remove individual *SNORD116* groups and do not affect *MAGEL2* expression would help to determine if effects we observed are due in part to *MAGEL2* or directly from *SNORD116*.

The novel list of genes we have described holds promise for future studies. There are a number of fascinating genes we have consistently implicated in the disorder, like *PAX6* which may contribute to some of the vision phenotypes reported in PWS patients (82); *IRX5* which has been implicated in obesity and metabolism (83); and *FGF13* (formerly referred to as *FHF2*) which is contained within a region on the X chromosome where aberrations cause strikingly similar Prader-Willi-like phenotypes such as hypotonia, failure-to-thrive, developmental delay, intellectual disability, and in some cases reproductive system anomalies and obesity (84,85)). Most notably, however, is the consistent dysregulation of *MAGEL2*. Mutations in *MAGEL2* cause Schaaf-Yang syndrome (SYS), which shares some phenotypes with PWS (20) (https://www.ncbi.nlm.nih.gov/books/NBK567492/). Even more interesting is that *MAGEL2* is the only shared gene across the subset of previously mentioned studies we analyzed and this study. This may suggest that both *SNORD116* loss and *MAGEL2* dysregulation drive PWS phenotypes.

## Supplementary Material

gkae1129_Supplemental_Files

## Data Availability

Sequencing data are available at Gene Expression Omnibus (GEO) accession GSE232183. Signal tracks for these experiments are available at the UCSC Genome Browser as a public session (https://genome.ucsc.edu/s/rbgilmore/PWS_RNAseq_bigwigs). All original code is publicly available on GitHub (https://github.com/cotneylab/SNORD116_targets_functions) and archived on Zenodo (10.5281/zenodo.13355488) (86). Any additional information required to reanalyze the data reported in this paper is available from the corresponding author upon request. Cell lines are available upon reasonable request and after completion of Material Transfer Agreements through the University of Connecticut Cell and Genome Engineering Core.

## Notes

Present address: Michael S. Chung, Department of Radiology, Emory University School of Medicine, Atlanta, GA, 30322, USA.

Present address: Rachel B. Gilmore, Institute for Human Genetics, Heidelberg University Hospital, Heidelberg, BW, 69120, Germany.
