# Supplementary material for: Identifying key underlying regulatory networks and predicting targets of orphan C/D box SNORD116 snoRNAs in Prader–Willi syndrome
Source: Nucleic Acids Res. 2024 Nov 22;52(22):13757–74. doi: 10.1093/nar/gkae1129 (PMC11662933; doi:10.1093/nar/gkae1129)
Supplement: gkae1129_Supplemental_Files [file gkae1129_supplemental_files.zip › Combined_Supplement_reduced.pdf]

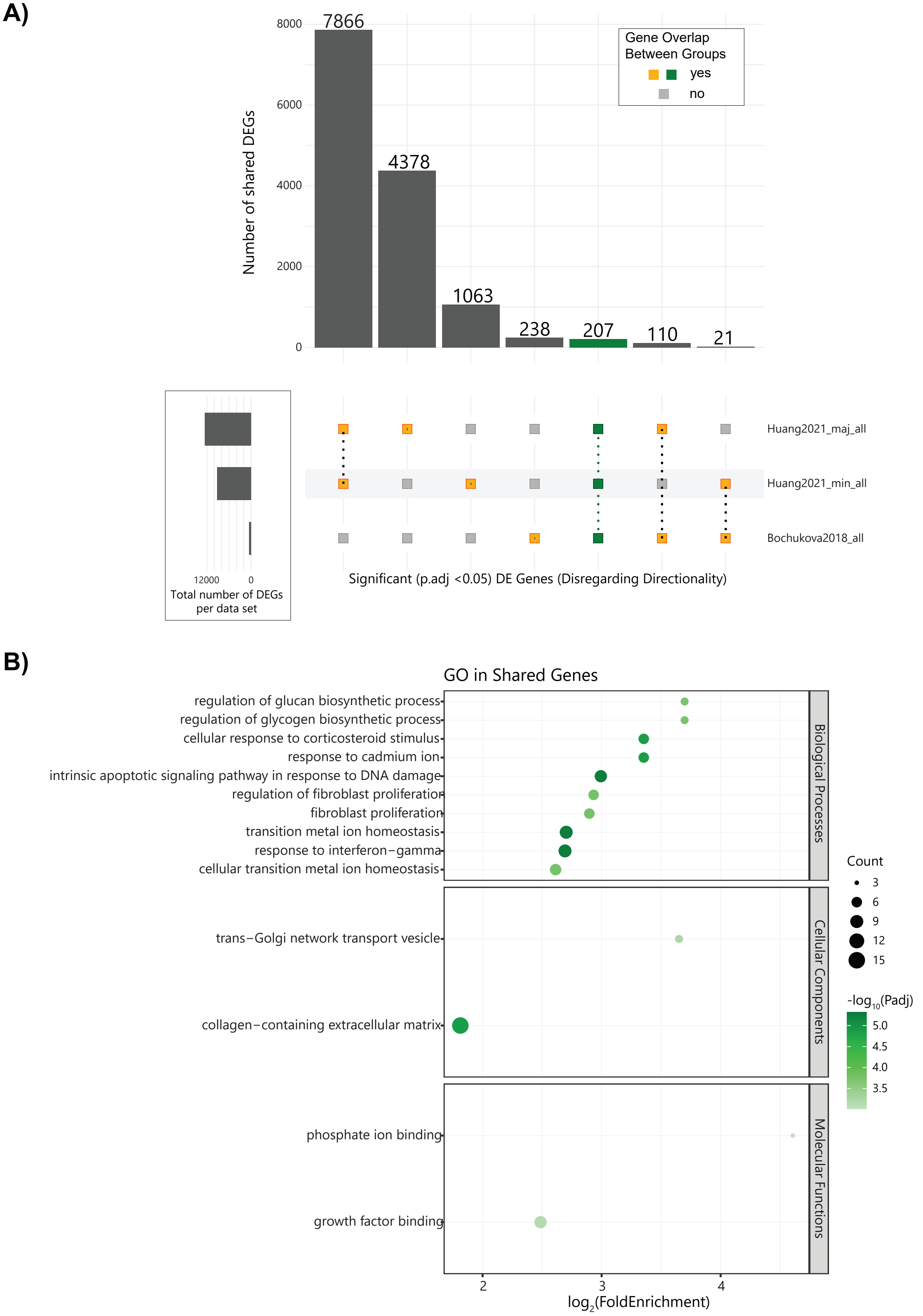

Supplemental Figure 1. A) Upset plot comparing significant DEGs (p.adjust < 0.05) in previously published studies. Green bar represents significant shared DEGs across all three data sets. **B)** Dot plot displaying gene ontology (GO) results for 207 shared dysregulated genes in all three studies. The x-axis represents the $\log_2$ fold enrichment value, and y-axis shows ontology terms. Size of the dot corresponds to the number of DEGs in our data set contained within each ontology term. Shading of the dot corresponds to the negative $\log_{10}$ of the adjusted p-value.

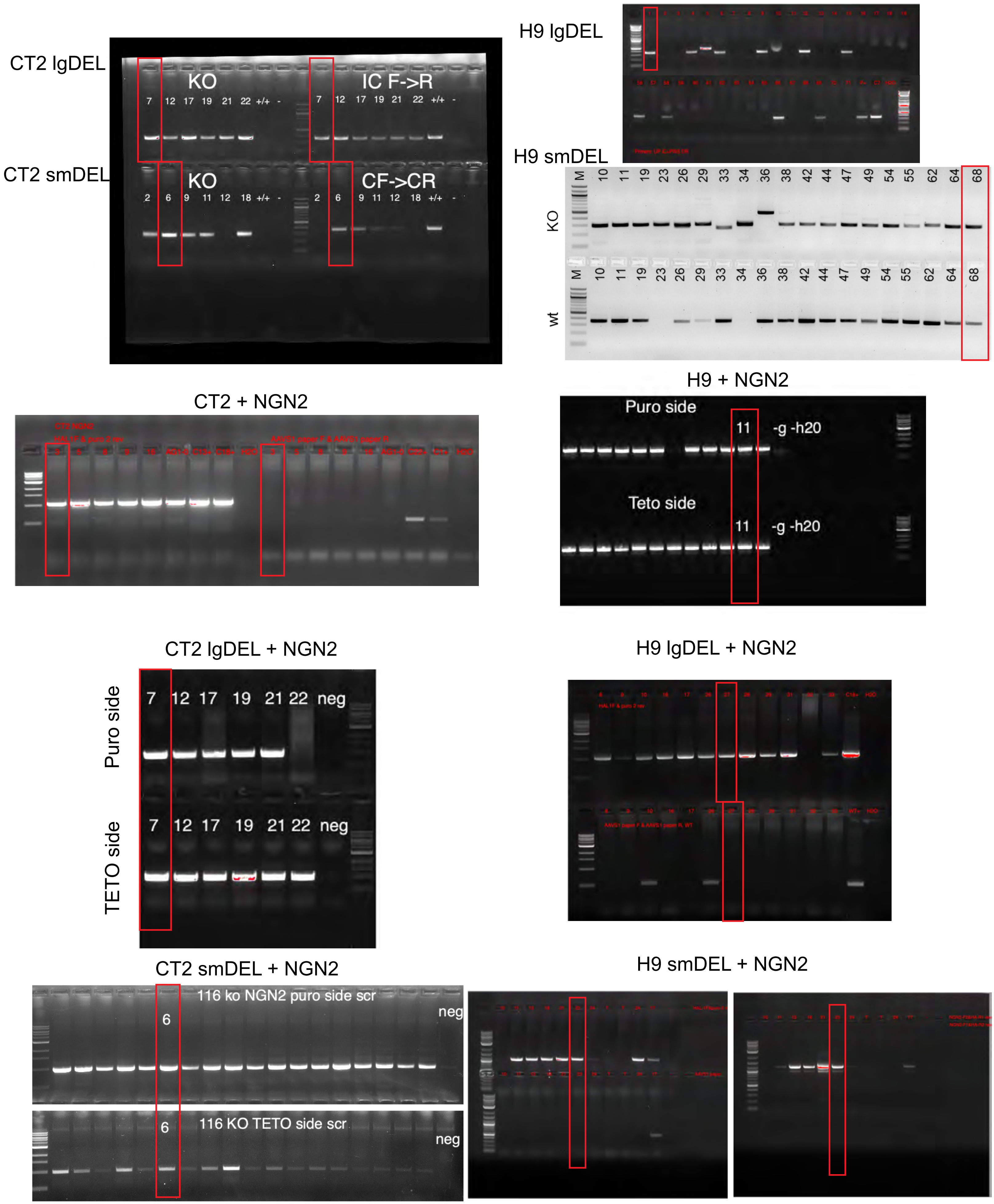

Supplemental Figure 2. Representative gel images from PCR screening clones for various genomic edits. Red boxes indicate clones that were used in sequencing experiment.

5’-IgDEL CRISPR

Guide: GTTAACCGCAGTATGAGTCA

Off-target: aTTtACCaCAGTATGAGTCATGG

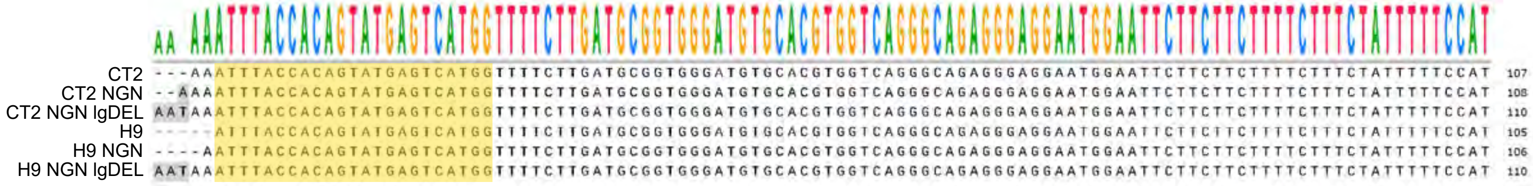

5’-smDEL CRISPR

Guide: TGGCTAGGTACACCTCACCT

Off-target #1: TGGCTAGGcAgACCTCACCcAGG

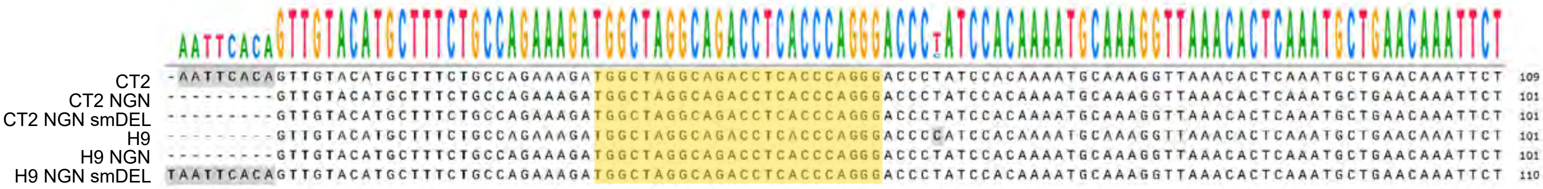

Guide: TGGCTAGGTACACCTCACCT

Off-target #2: TaGCgAGGTACACCTCACCaAGG

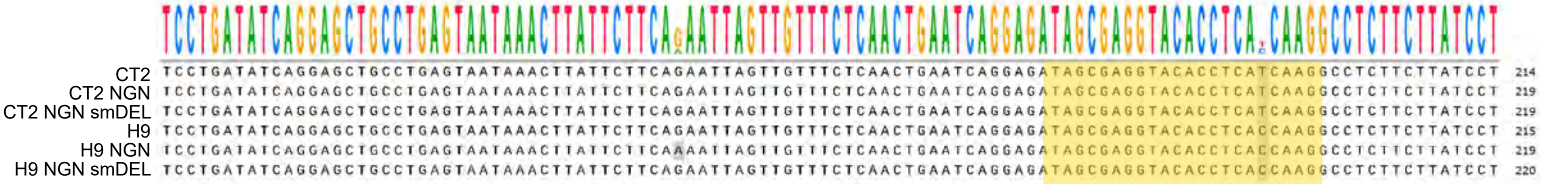

3’-DEL CRISPR

Guide: ATCGGCATGAACCAAGAACT

Off-target #1: ATCaGCATGAAgCcAGAACTGGG

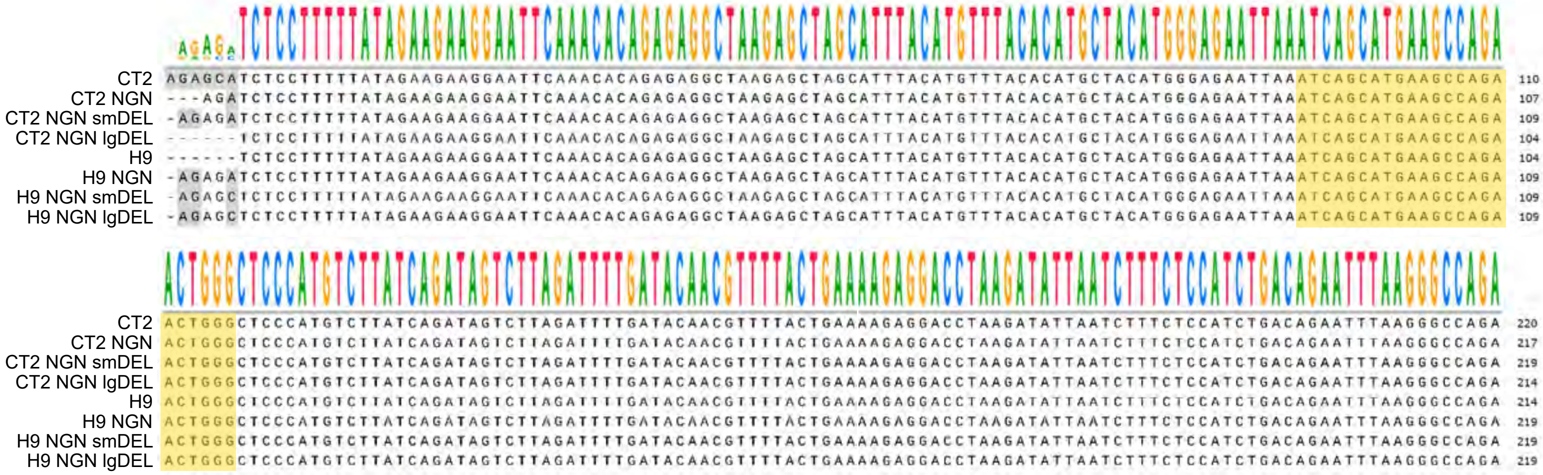

Guide: ATCGGCATGAACCAAGAACT

Off-target #2: ATCtGCATGAgCCAAtAACTGGG

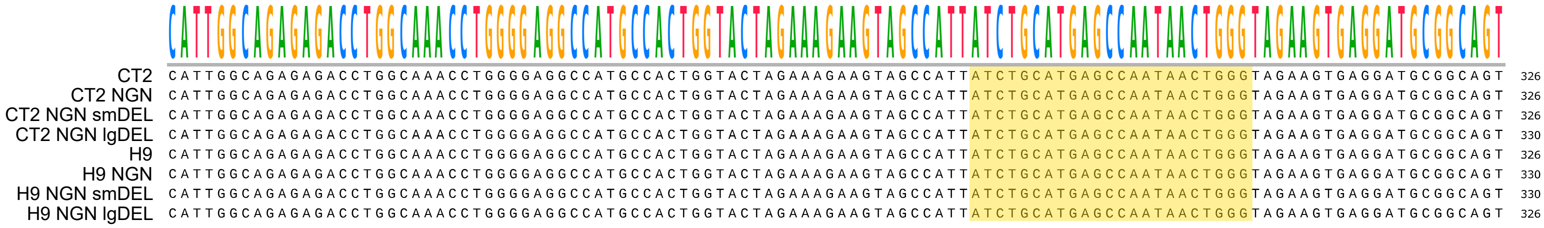

Supplemental Figure 3. Results from CRISPR off-target analysis. Sample name is to the left of the nucleotide sequence. Consensus sequence is shown above the alignment. Dashed line indicates missing bases, gray shading indicates a mismatch, and yellow shading indcates off-target sequence of interest. Differences in guide sequence compared to off-target sequence are shown in lowercase letters.

Illumina Infinium® CytoSNP-850K Assay Results

March 27, 2024

Justin Cotney, PhD
Rachel Gilmore
Samples submitted: February 23, 2024

RESULTS:

1. **H9 NGN2:**

   683.2 kb GAIN on CHROMOSOME 7
   - BAND: 7q11.21-7q11.21
   - POSITION: 62,015,939-62,699,114

   108.3kb LOSS on CHROMOSOME 19
   - Note this is below our level of detection (400kb). It is reported because this was an area of interest.
   - BAND: 19q13.42-19q13.42
   - POSITION: 55,515,784-55,624,113

2. **H9 NGN2 SNORD116 (smDEL):**

   713.5 kb GAIN on CHROMOSOME 7
   - BAND: 7q11.21-7q11.21
   - POSITION: 61,985,583-62,699,114

3. **H9 NGN2 SNORD116 IC (lgDEL):**

   652.0 kb GAIN on CHROMOSOME 7
   - BAND: 7q11.21-7q11.21
   - POSITION: 62,047,108-62,699,114

   335.0 kb LOSS on CHROMOSOME 15
   - Note this is below our level of detection (400kb). It is reported because this was an area of interest.
   - BAND: 15q11.2-15q11.2
   - POSITION: 25,016,528-25,351,551

4. **CT2 NGN2:**

   Apparently Normal Female

5. **CT2 NGN2 SNORD116 (smDEL):**

   Apparently Normal Female

6. **CT2 NGN2 SNORD116 IC (lgDEL):**

   335.0 kb LOSS on CHROMOSOME 15
   - Note this is below our level of detection(400kb). It is reported because this was an area of interest.
   - BAND: 15q11.2-15q11.2
   - POSITION: 25,016,528-25,351,551

7. **CT2 (parent line/unedited):**

   Apparently Normal Female

BeadChip v1.2:
Samples 1-5: 207521840012
Samples 6-7: 207521840026
Call Minimums:
Gains/Losses: 400Kb
LOH: 5Mb

Supplemental Figure 4. Results from CytoSNP Array on all edited cell lines. Gain on chromosome 7 in H9 lines is consistent with previous CytoSNP array analysis of parental H9 cell line used in the lab.

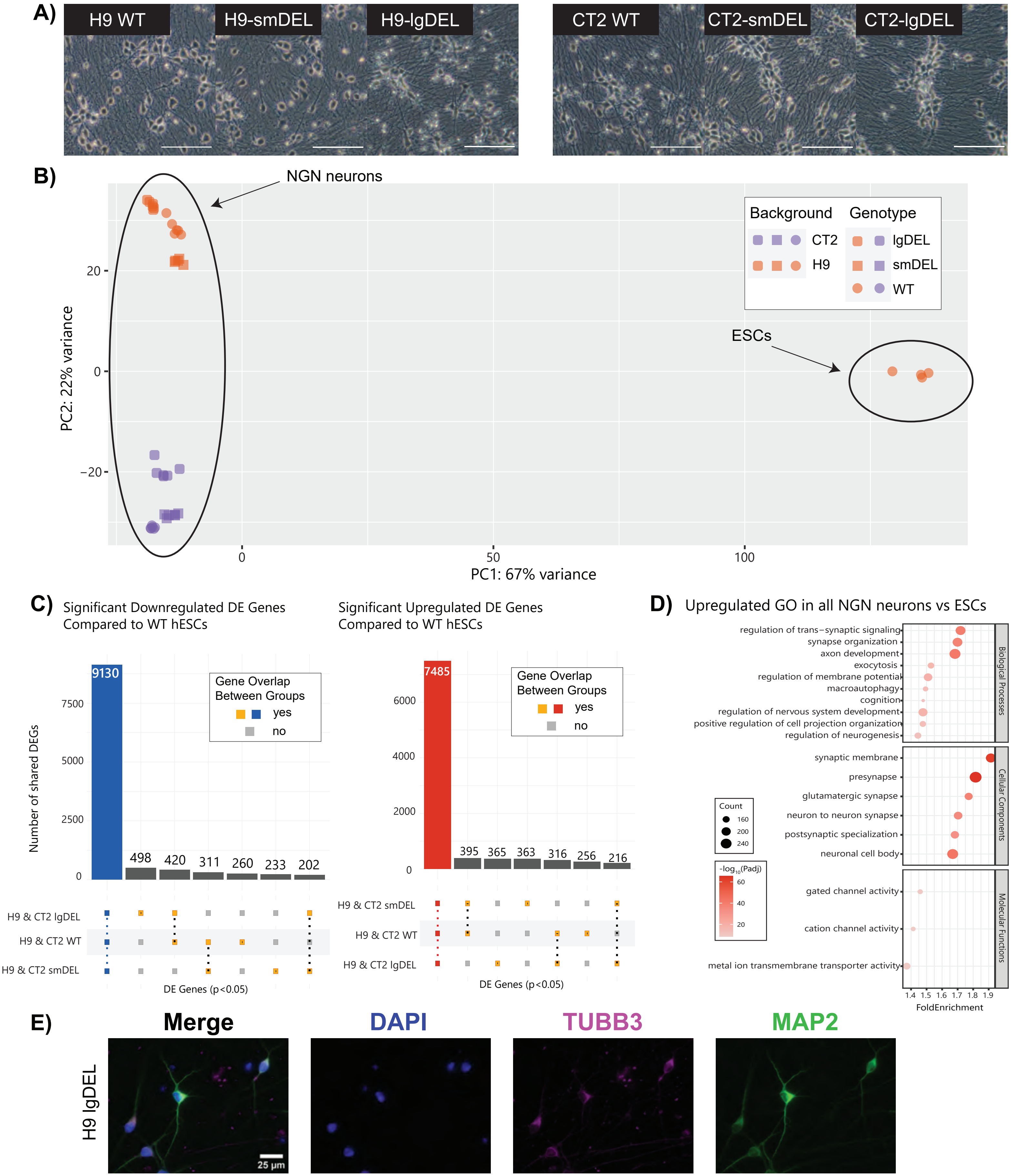

Supplemental Figure 5. A) Representative brightfield images of neurons taken at 20x magnification, scale bar is equal to 100 μm. **B)** PCA plot displaying variance of samples. Individual samples colored by background. Shapes indicate genotype. Lineage is denoted by circle with label. **C)** Upset plots comparing significant DEGs (p.adjust < 0.05) of wild type (WT), smDEL, and lgDEL inducible neurons across both genetic backgrounds to wild type H9 ESCs. Blue bar represents significant shared downregulated (log_2FoldChange < 0) DEGs and red bar represents significant shared upregulated (log_2FoldChange > 0) DEGs in all three genotypes versus WT ESCs. **D)** Dot plot displaying gene ontology (GO) results for 7485 shared upregulated DEGs in C. The x-axis represents the fold enrichment value, and y-axis shows ontology terms. Size of the dot corresponds to the number of DEGs in data set contained within each ontology term. Shading of the dot corresponds to the negative log_{10} of the adjusted p-value. **E)** Representative immunocytochemistry images of H9 lgDEL neurons taken at 63x magnification.

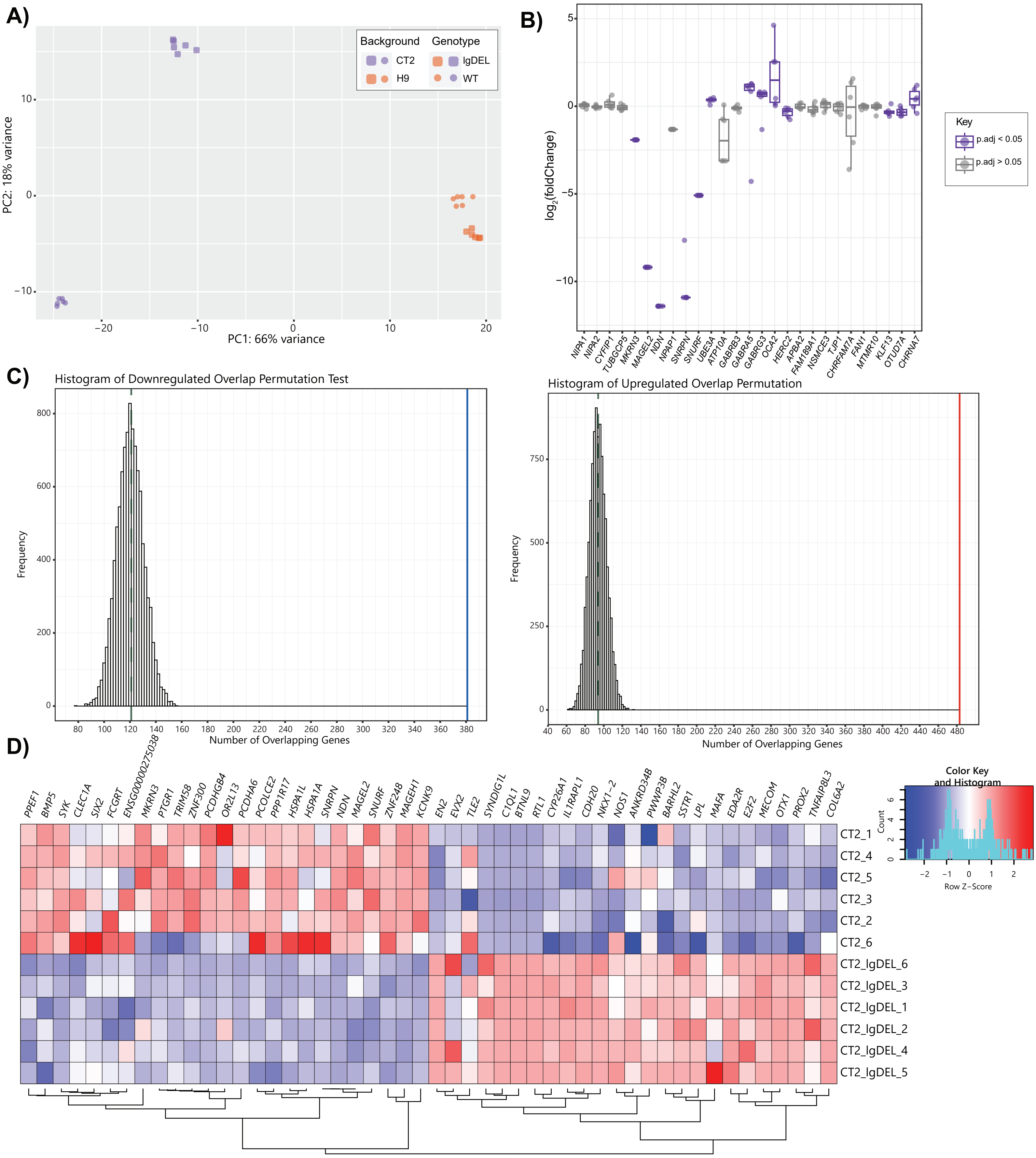

Supplemental Figure 6. A) PCA plot displaying variance of NGN neuron samples. Individual samples colored by background. Shapes indicate genotype. **B)** Box and whisker plot showing differential expression of protein-coding genes in the chromosome 15q11-q13 region for CT2 IgDEL line vs WT. Pseudocount was added to counts of all genes prior to calculation of $\log_2$(foldchange). Significant DEGs (p.adjust < 0.05) are shown in purple. Not all protein-coding genes shown for simplicity. **C)** Histogram of permutation test for overlapping genes. Green dashed line represents median number of overlaps. Solid blue bar (*left*) represents number of shared downregulated genes. Solid red line (*right*) represents number of shared upregulated genes. **D)** Heatmap showing 50 most dysregulated significant DEGs in IgDEL vs WT CT2 genetic background. Top 25 up- and downregulated genes were determined by average log2(foldChange) between CT2 and H9 backgrounds. Shading indicates row z-score, with blue denoting downregulated gene expression and red denoting upregulated gene expression. Rows represent samples; columns represent individual genes.

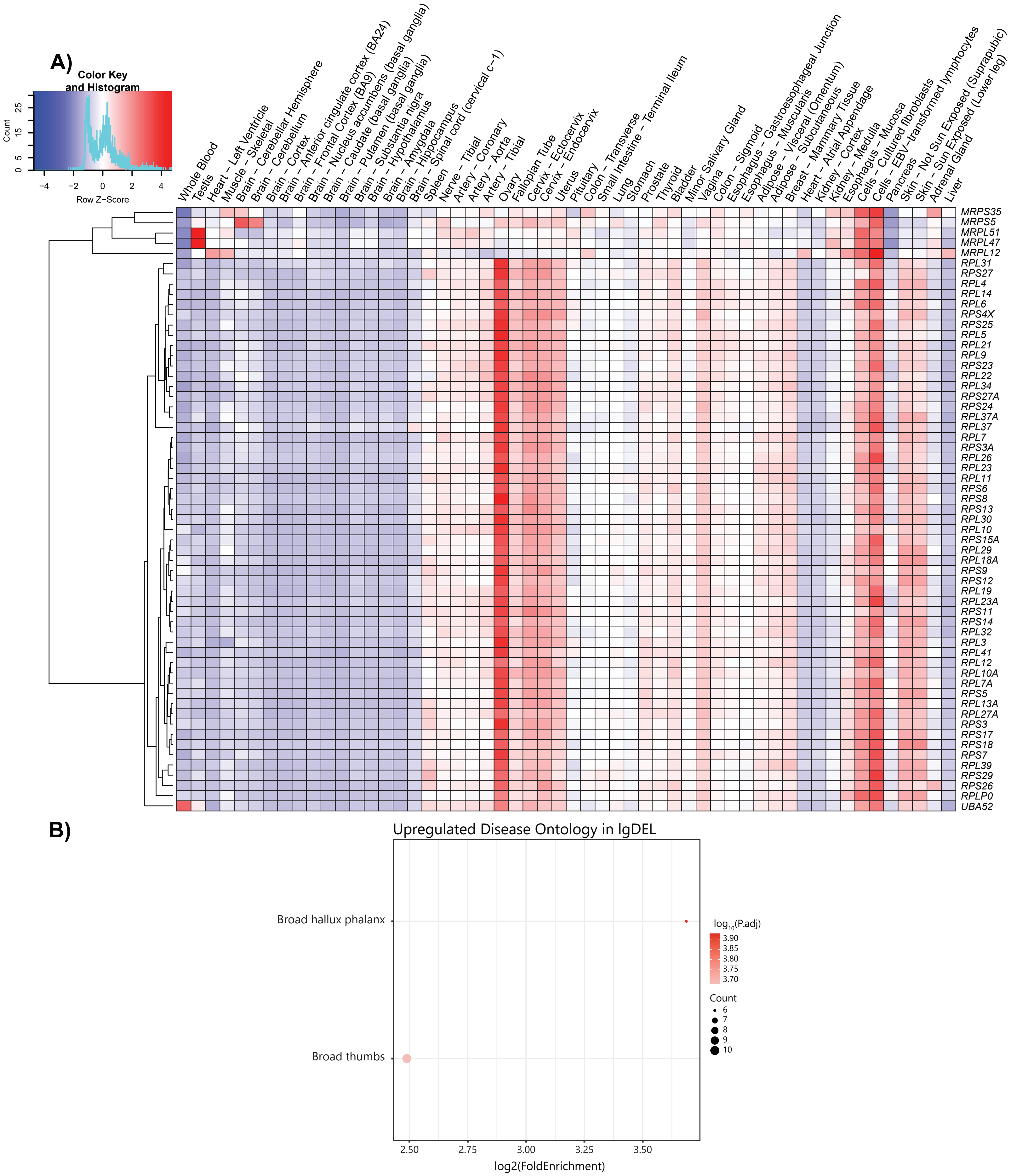

Supplemental Figure 7. A) Heatmap of expression of significant DEGs contained within the structural constiuent of the ribosome ontology (GO0003735) in GTEx tissues. The x-axis displays the tissue type from which expression was profiled, and the y-axis displays the gene name. Shading of the graph corresponds to row z-score. Red indicates higher comparative gene expression and blue indicates lower comparative gene expression. **B)** Dot plot displaying disease ontology results for shared downregulated genes. The x-axis represents the $\log_2$ fold enrichment value, and y-axis shows disease ontology terms. Size of the dot corresponds to the number of DEGs in our data set contained within each ontology term. Shading of the dot corresponds to the negative $\log_{10}$ of the adjusted p-value.

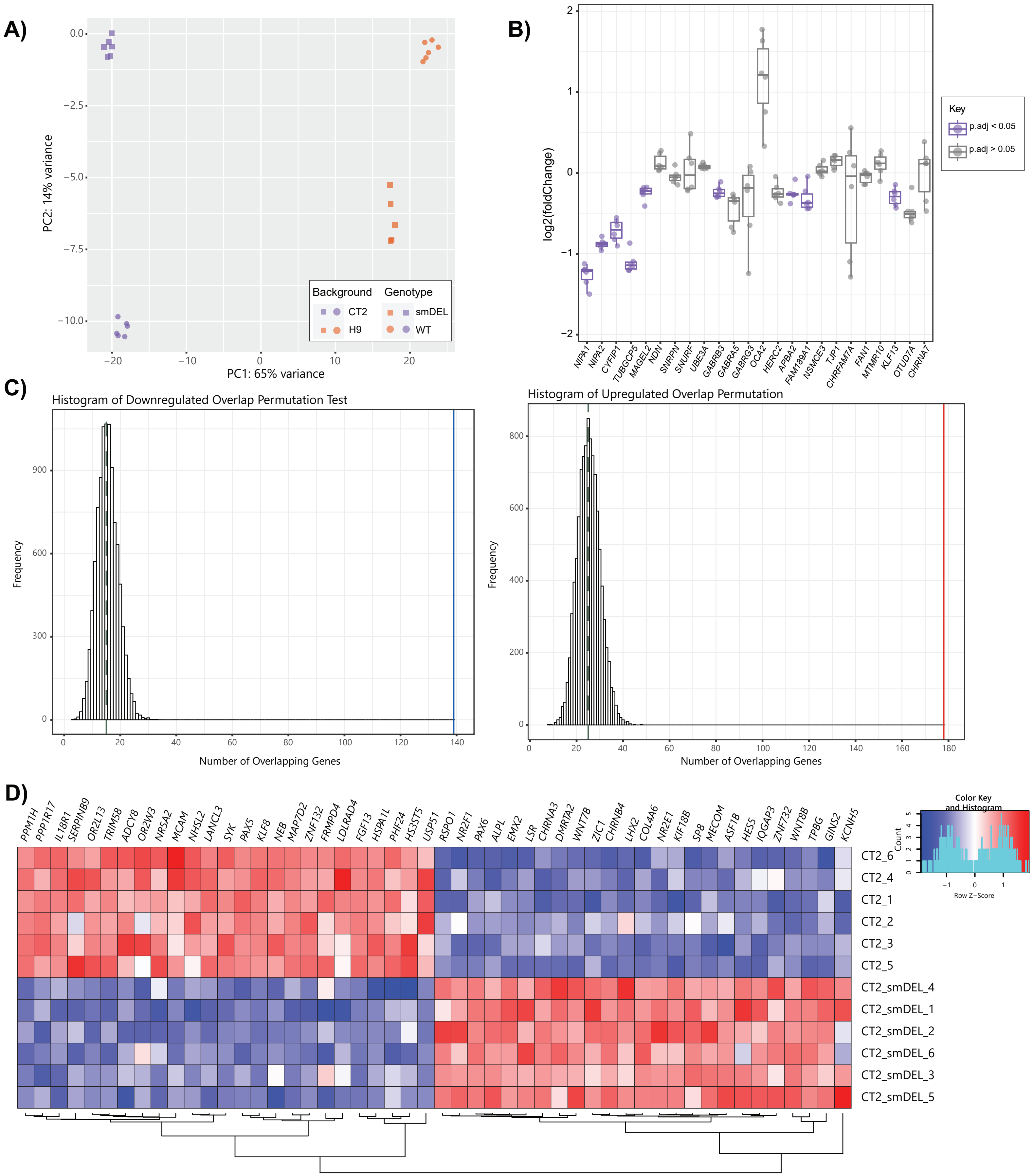

Supplemental Figure 8. A) PCA plot displaying variance of NGN neuron samples. Individual samples colored by background. Shapes indicate genotype. **B)** Box and whisker plot showing differential expression of protein-coding genes in the chromosome 15q11-q13 region for CT2 smDEL line vs WT. Pseudocount was added to counts of all genes prior to calculation of log_2(foldchange). Significant DEGs (p.adjust < 0.05) are shown in purple. Not all protein-coding genes shown for simplicity. **C)** Histogram of permutation test for overlapping genes. Green dashed line represents median number of overlaps. Solid blue bar (*left*) represents number of shared downregulated genes. Solid red line (*right*) represents number of shared upregulated genes. **D)** Heatmap showing 50 most dysregulated significant DEGs in smDEL vs WT CT2 genetic background. Top 25 up- and downregulated genes were determined by average log2(foldChange) between CT2 and H9 backgrounds. Shading indicates row z-score, with blue denoting downregulated gene expression and red denoting upregulated gene expression. Rows represent samples; columns represent individual genes.

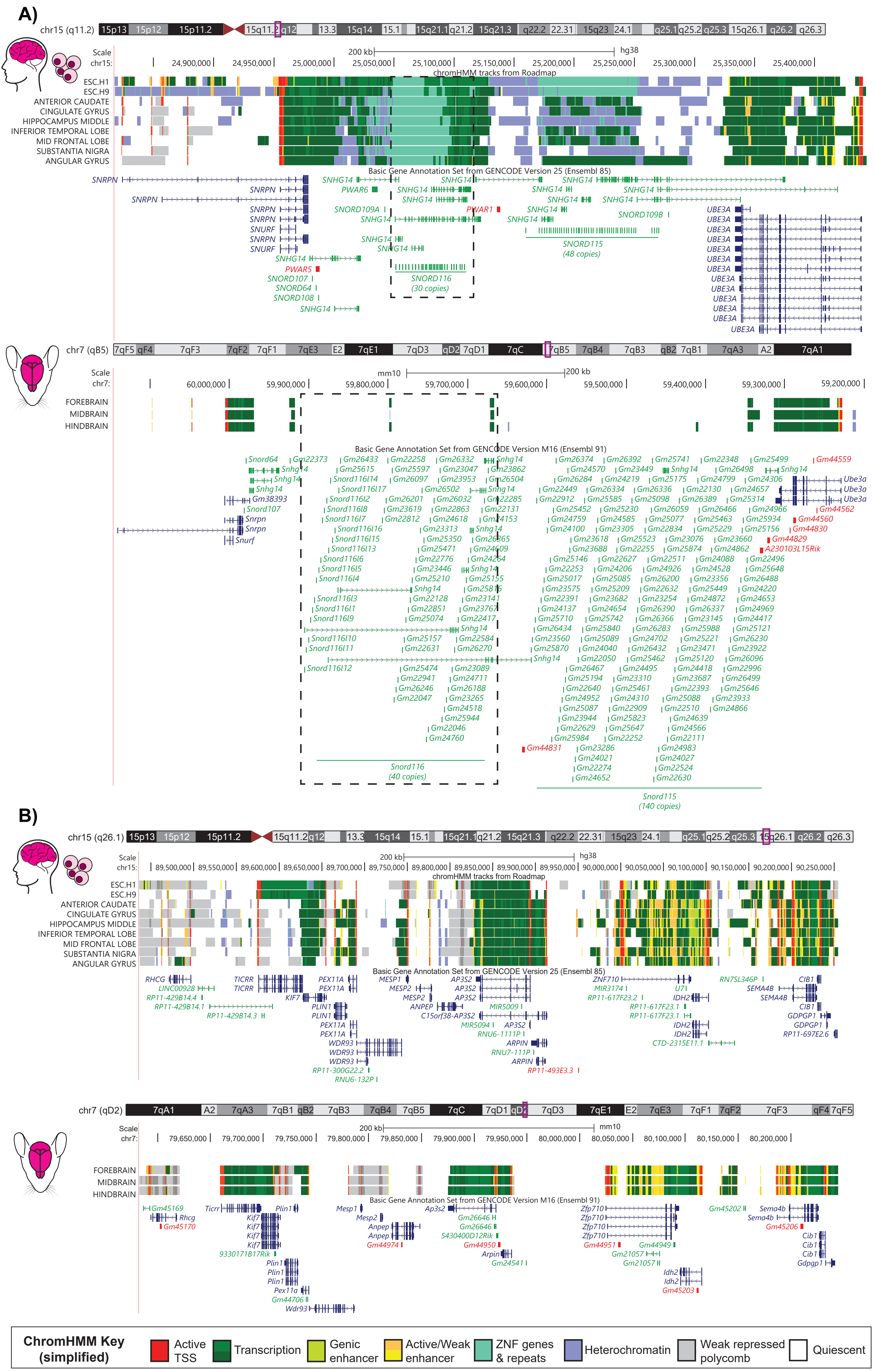

Supplemental Figure 9. UCSC Browser Images of human and mouse loci for A) PWS locus and B) another syntenic locus. ChromHMM 18-state (auxiliary) tracks for human are from various brain regions and ESCs; data was generated through Roadmap Epigenome. ChromHMM 18-state (auxiliary) tracks for mouse are from postnatal day 0 mice brain regions and were generated by the Cotney lab. GENCODE gene annotations can be found under ChromHMM tracks. Black dashed box highlights *SNORD116/Snord116* region.

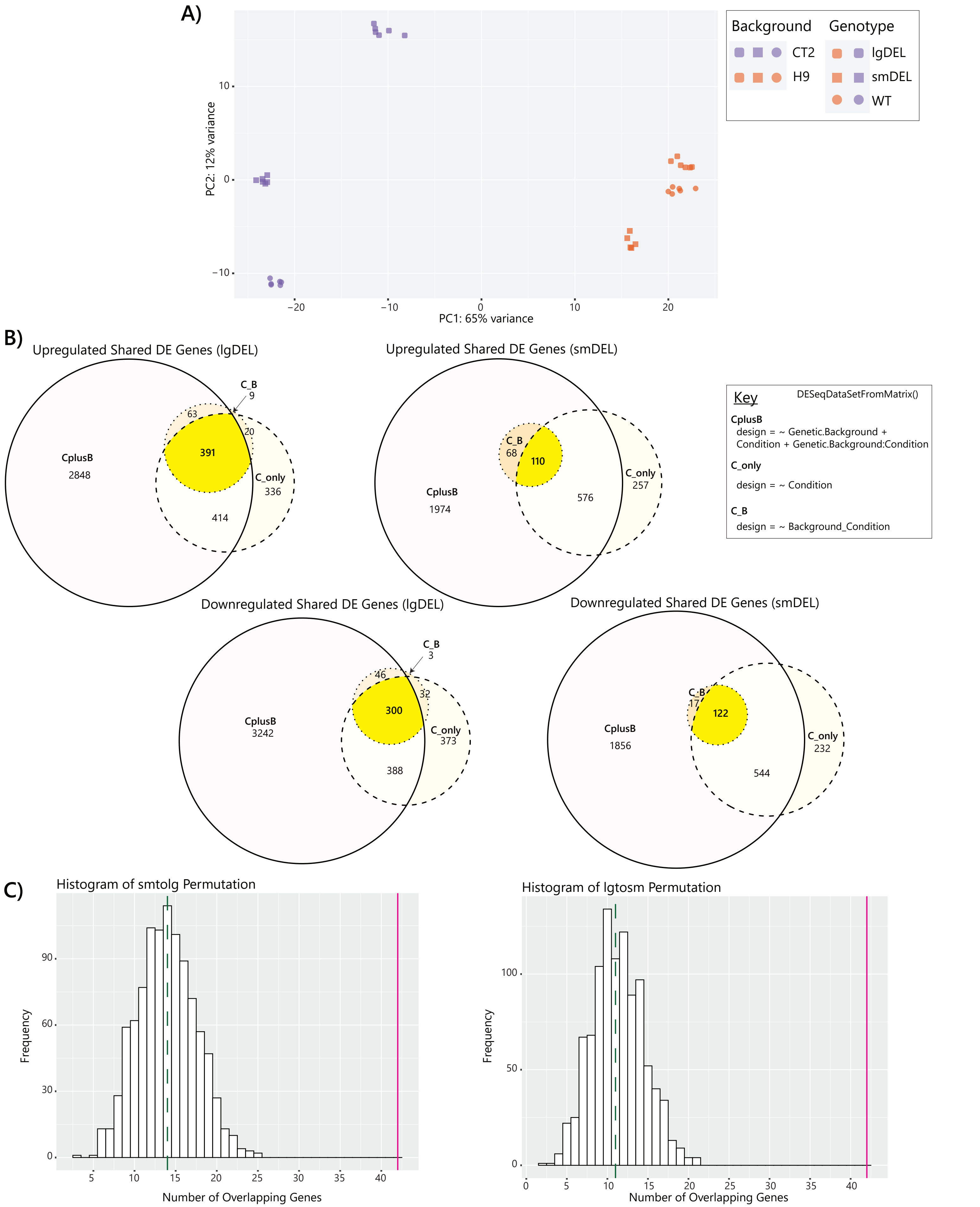

Supplemental Figure 10. A) PCA plot displaying variance of samples. Individual samples colored by background. Shapes indicate genotype. **B)** Venn diagrams showing overlap of significant DEGs (p.adjust < 0.05) of three separate DESeqDataSetFromMatrix() designs for lgDEL (top left, bottom left) and smDEL (top right, bottom right). Bright yellow shading represents significant shared DEGs across all three analyses. **C)** Histogram of permutation test for overlapping genes. Green dashed line represents median number of overlaps in either the permutation of overlaps in smDEL to lgDEL datasets (*left*) or lgDEL to smDEL datasets (*right*). Solid pink bar represents 42 shared dysregulated genes.

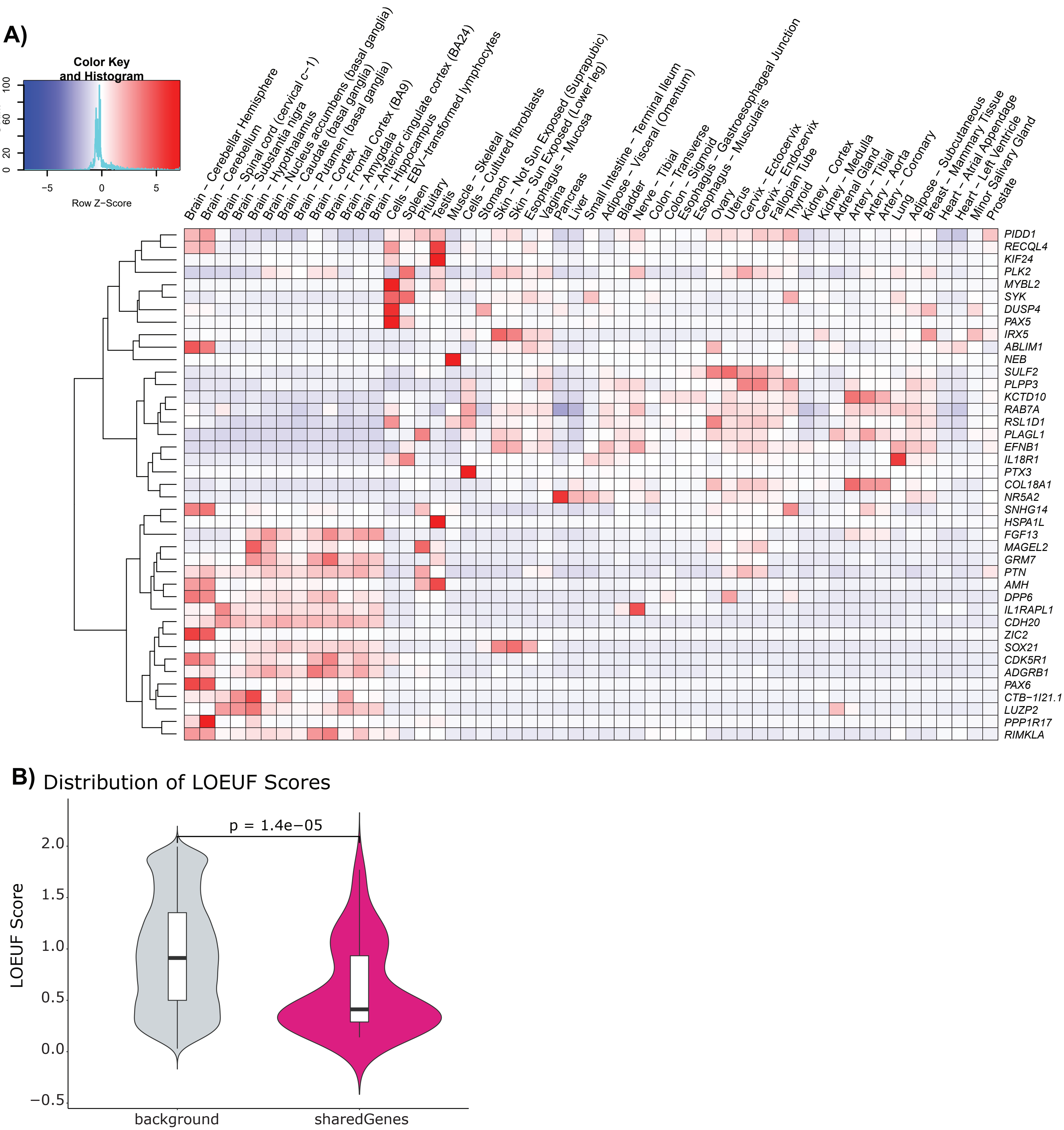

Supplemental Figure 11. A) Heatmap of expression of shared dysregulated genes in GTEx tissues. The x-axis displays the tissue type from which expression was profiled, and the y-axis displays the gene name. Shading of the graph corresponds to row z-score. Red indicates higher comparative gene expression and blue indicates lower comparative gene expression. **B)** Violin plot showing distribution of LOEUF scores of shared dysregulated genes (pink) compared to the rest of the gnomAD database (gray). Significance determined by Wilcoxon Rank Sum Test.

A)

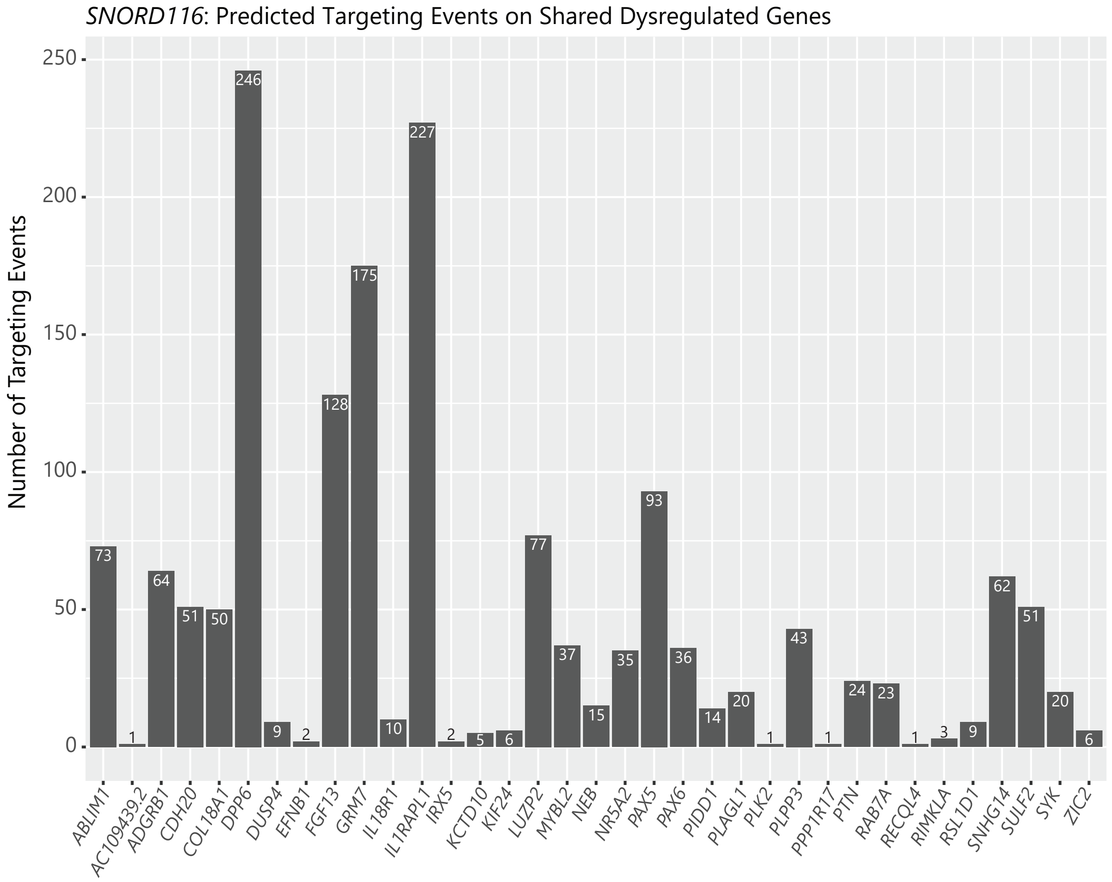

B)

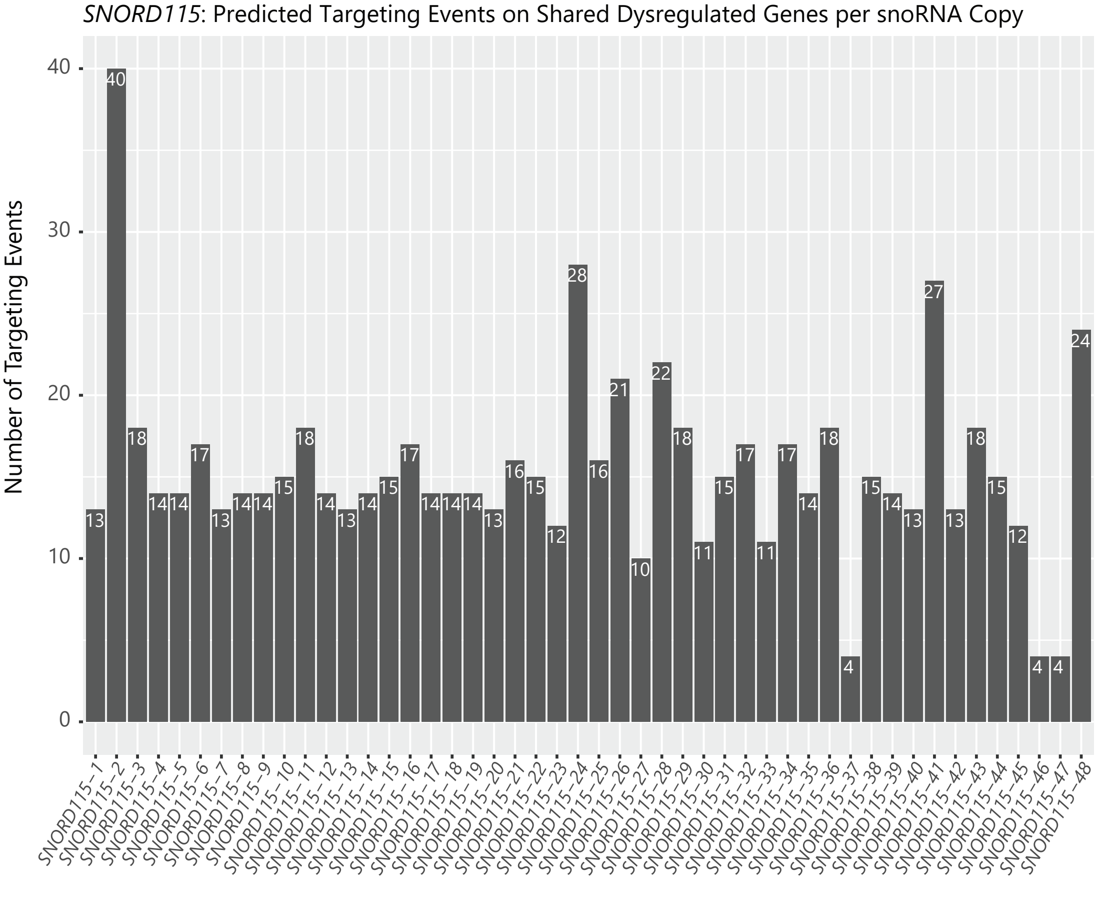

Supplemental Figure 12. A) Bar plot representing the number of predicted targeting events of *SNORD116* on shared list of dysregulated genes. The x-axis displays the predicted number of targeting events, and the y-axis displays the target gene names. Note that this excludes predicted targeting events from three additional *SNORD116* copies found on chromosomes other than chr15. **B)** Bar plot representing the number of predicted targeting events per copy of *SNORD115*. The x-axis displays the predicted number of targeting events, and the y-axis displays the *SNORD115* copies. Note that this excludes two additional copies of *SNORD115* found on chromosomes other than chr15.

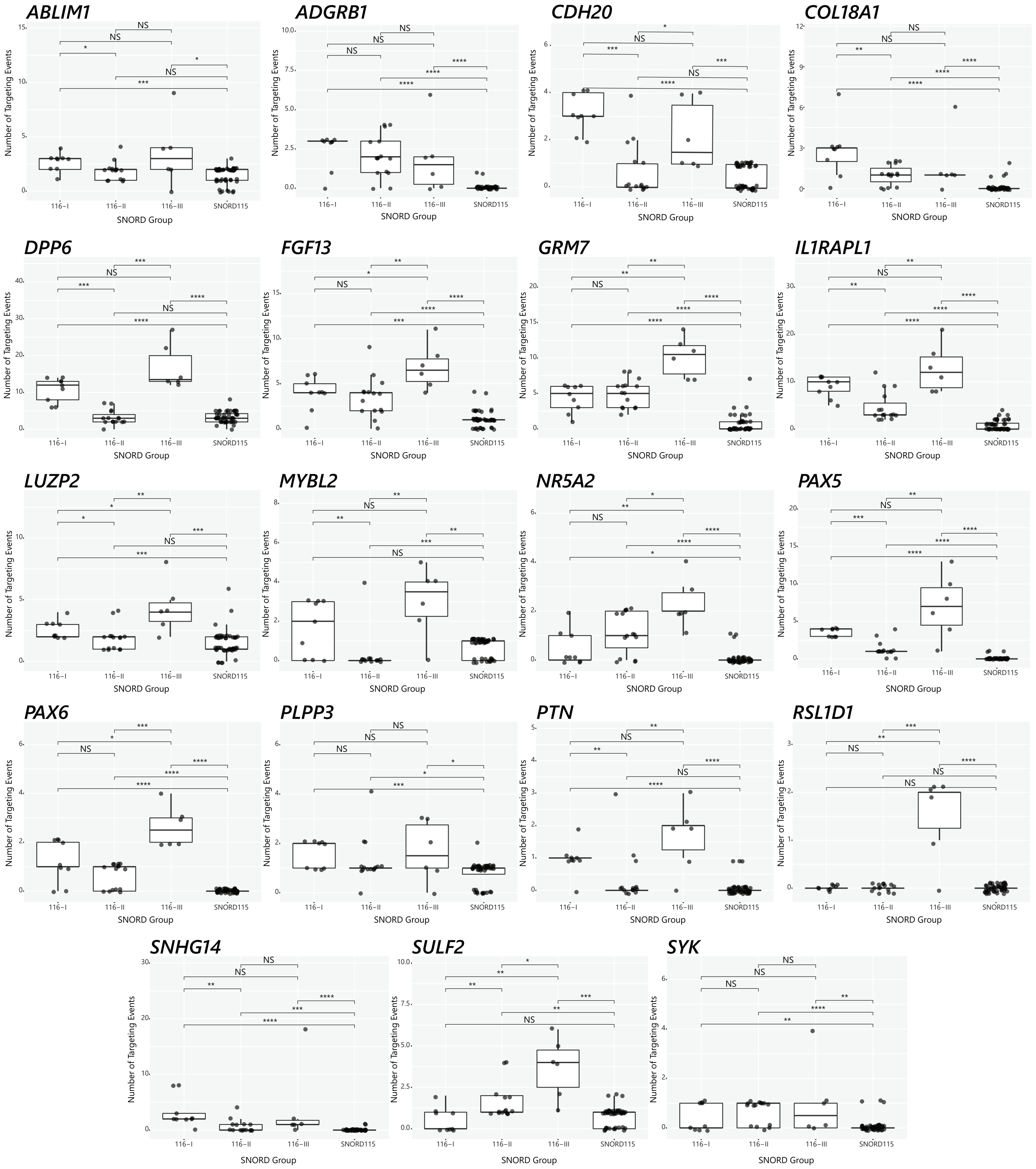

Supplemental Figure 13. Box and whisker plots displaying enrichment of *SNORD116* predicted targeting events per gene compared to *SNORD115*. The x-axis displaysthe SNORD group (*SNORD116-I*, *SNORD116-II*, *SNORD116-III*, and *SNORD115* from left to right). The y-axis represents the number of predicted targeting events. Each plot is an individual gene. Significance was determined by the Wilcoxon Test; NS = not significant (p-value > 0.05), * = p-value < 0.05, ** = p-value < 0.01, *** = p-value < 0.001, **** = p-value < 0.0001.

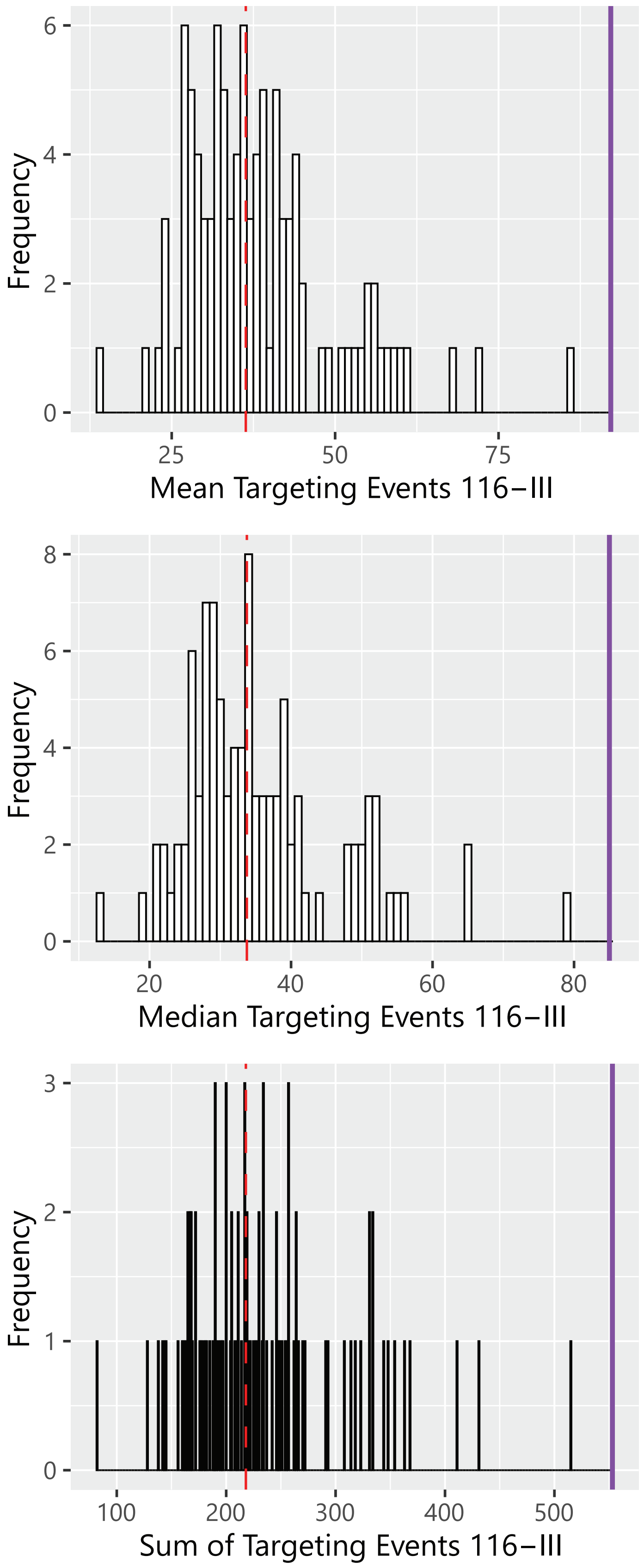

Supplemental Figure 14. Histogram of permutation test for predicted *SNORD116-III* targeting events on 100 random sets of 42 genes which did not differ significantly (via Wilcoxon test) from the set of 42 shared dysregulated genes in length, GC content, or expression in inducible neuron system. Red dashed line represents median number of mean (*top*), median (*middle*), or sum (*bottom*) of predicted targeting events. Solid purple bar represents number of experimentally obtained mean (*top*), median (*middle*), or sum (*bottom*) of predicted *SNORD116-III* targeting events on shared dysregulated genes.

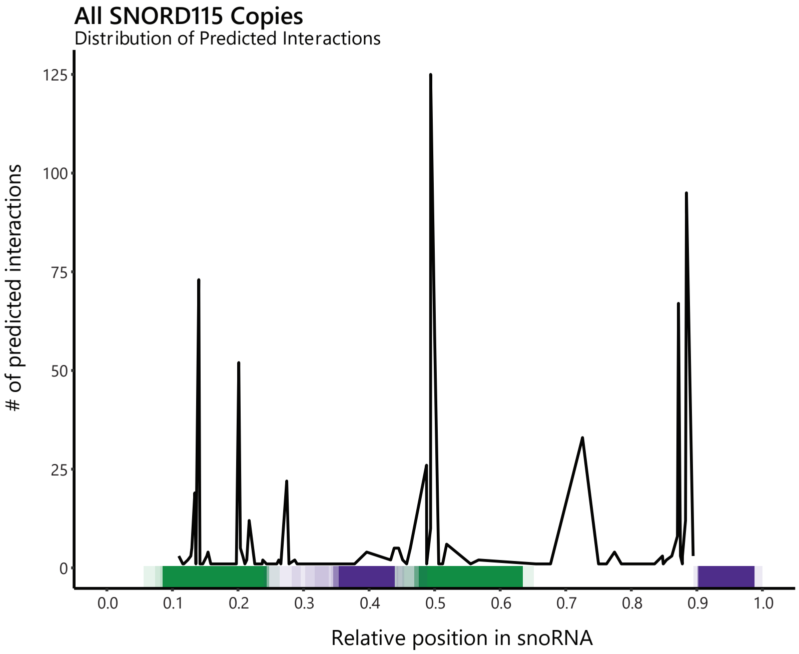

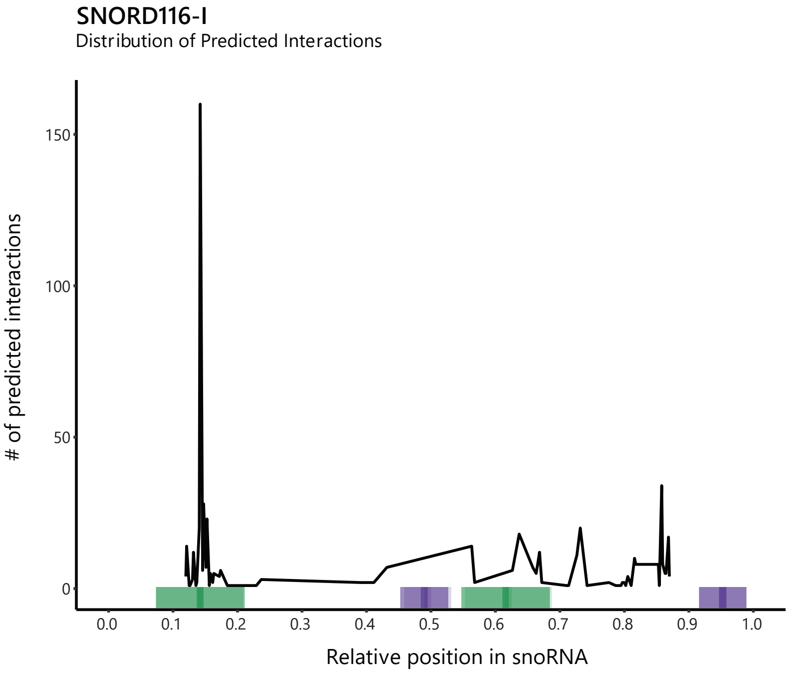

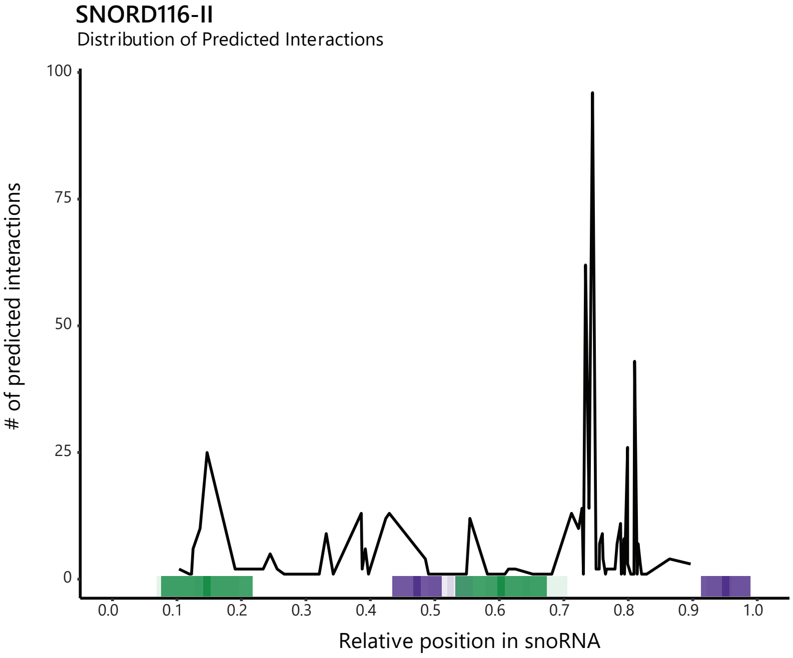

Supplemental Figure 15. Plots displaying distribution of prediction interactions for *SNORD115* (*top*), *SNORD116-I* (*bottom left*), and *SNORD116-II* (*bottom right*). The x-axis corresponds to the relative position within snoRNA copies, and y-axis represents the number of predicted interactions for which the center of the predicted binding interaction was used (black line). Color-coded bar on the x-axis indicates the position of C/C' and D/D' boxes found in snoRNA copies, indicated by green and purple respectively. The canonical antisense elements (ASEs) are found upstream of D/D' boxes.

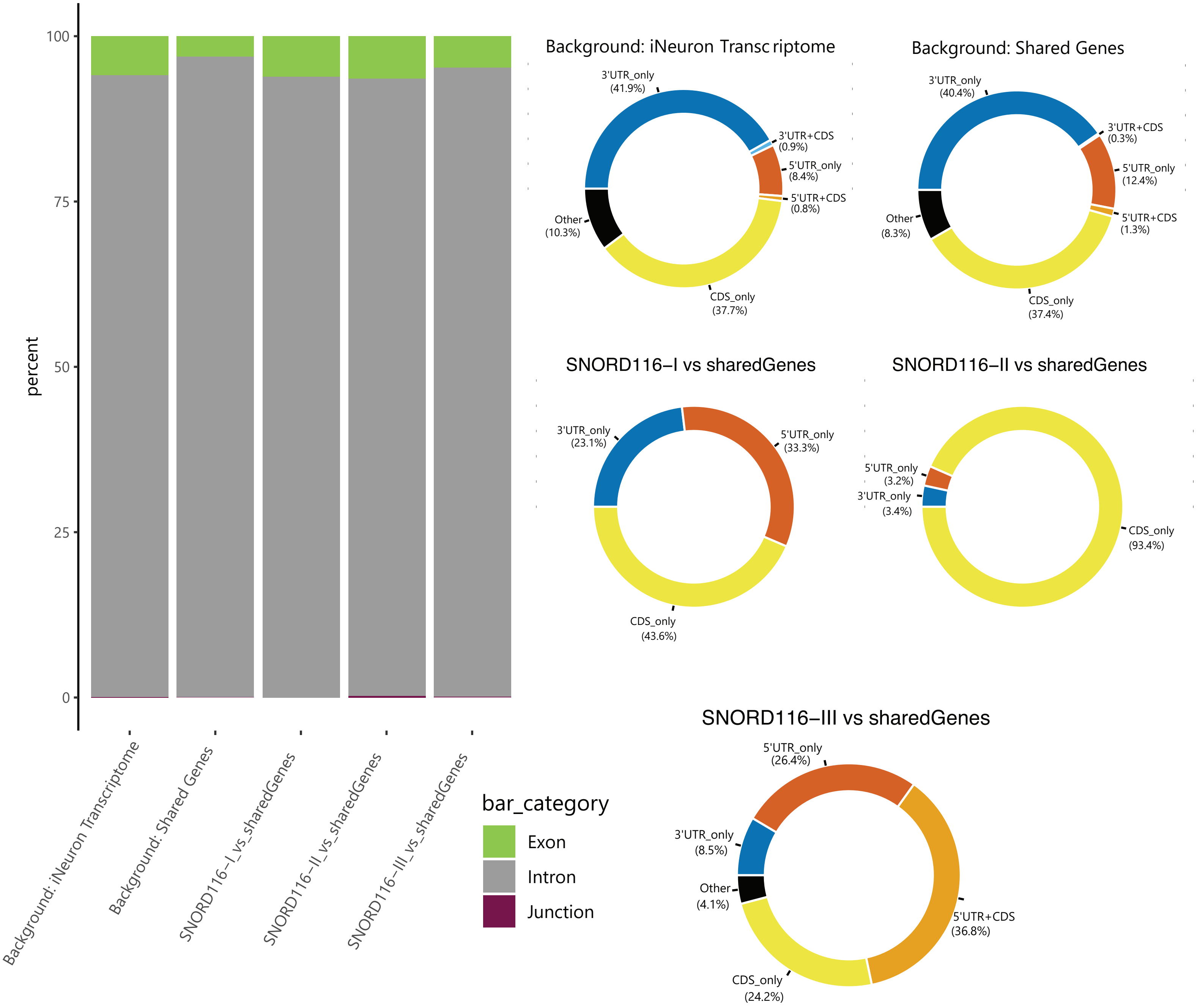

Supplemental Figure 16. Bar charts representing the proportion of exon, intron, and intron-exon junctions in the entire inducible neuron transcriptome, the set of 42 shared dysregulated genes, and the predicted targeting of *SNORD116-I*, *SNORD 116-II*, and *SNORD116-III* copies on those shared genes (*SNORD116-I/II/III* vs Shared Genes). Exon category is subdivided based on genic location and displayed as donut plots. Coloring of donut plots is based on exon category; 5'UTRs are represented in orange, 3'UTRs are represented in blue, CDS is represented in yellow, and any portion of exonic sequence not falling under those categories is termed "other" and shown in black.

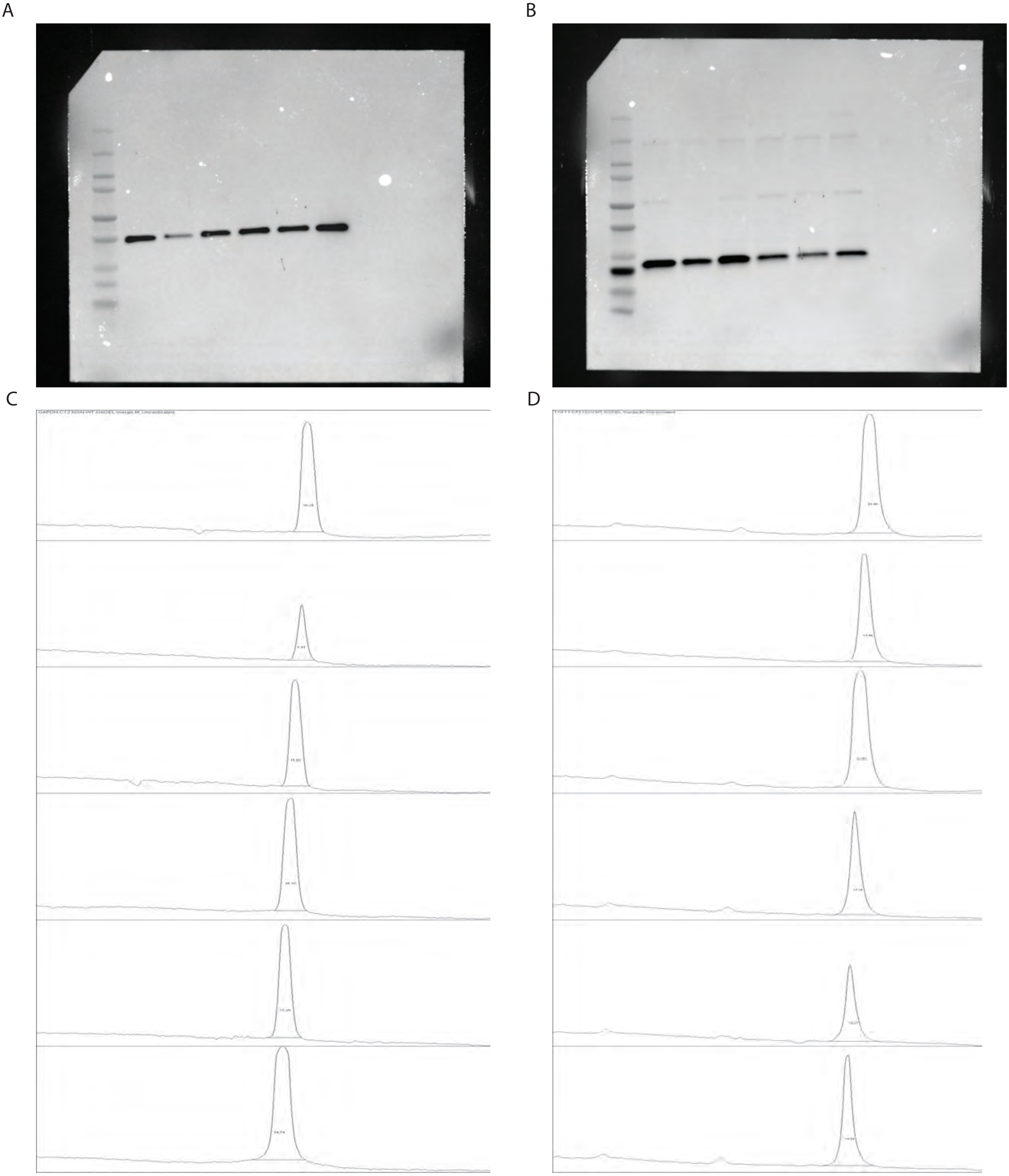

Supplemental Figure 17. Images of full western blot with quantification. Merged images of blots showing chemiluminescent bands for either A) housekeeping control GAPDH or B) FGF13 and the colorimetric ladder (lane 1). Lanes 2-4 are CT2 WT and 5-7 are CT2 smDEL. Area under the curve plots for C) GAPDH or D) FGF13. Lanes 2-7 are shown top to bottom, respectively.
